# Systematic review and meta-analysis of cardiac complications in acute pancreatitis

**DOI:** 10.1016/j.isci.2025.114172

**Published:** 2025-12-04

**Authors:** Veronika Lillik, Mahmoud Obeidat, Dániel Sándor Veres, Péter Ferdinandy, Elizabet Bodó, Asal Pourrastegar, Ali Moradi, Péter Hegyi, Rita Nagy

**Affiliations:** 1 Centre for Translational Medicine, Semmelweis University, Budapest, Hungary; 2 Szent György Teaching Hospital of Fejér County, Székesfehérvár, Hungary; 3 Department of Biophysics and Radiation Biology, Semmelweis University, Budapest, Hungary; 4 Center for Pharmacology and Drug Development, Semmelweis University, Budapest, Hungary; 5 Department of Pharmacology and Pharmacotherapy, Semmelweis University, Budapest, Hungary; 6 Pharmahungary Group, Szeged, Hungary; 7 Institute of Pancreatic Diseases, Semmelweis University, Budapest, Hungary; 8 Institute for Translational Medicine, Medical School, University of Pécs, Pécs, Hungary; 9 Translational Pancreatology Research Group, Interdisciplinary Centre of Excellence for Research Development and Innovation, University of Szeged, Szeged, Hungary; 10 Heim Pál National Pediatric Institute, Budapest, Hungary

**Keywords:** Cardiovascular medicine, Human metabolism, Public health

## Abstract

Cardiovascular abnormalities are associated with acute pancreatitis (AP). Here, we temporarily link these abnormalities to AP outcomes. We systematically searched PubMed, EMBASE, and Cochrane on 5^th^ November 2023 and included 44 studies (*n* = 5.3 million patients, 63.8% retrospective) containing United States National Inpatient Sample cohorts, where overlap may occur; without these databases, 1.2 million patients remained. We analyzed chronic heart failure (CHF), atrial fibrillation (AF), repolarization changes, and QTc prolongation on ECG in association with mortality and severity using odds ratios (ORs) and mean differences (MDs) with 95% confidence intervals (CIs) in a random-effects model. AP patients with AF face 2.69-fold increased odds of mortality in AP (OR, 2.69; CI, 1.34–5.38). Severe and moderately severe AP ([M]SAP) is associated with 2.75-times higher odds of repolarization changes on ECG (OR, 2.75; CI, 1.19–6.36). Cardiac assessment is essential in acute pancreatitis; prospective studies should address long-term cardiovascular complications.

## Highlights

• Pre-existing heart failure increases the odds of mortality in acute pancreatitis
• Atrial fibrillation triples the odds of death among acute pancreatitis patients
• Severe acute pancreatitis is linked to ECG repolarization abnormalities
• Cardiologic screening should be included in acute pancreatitis management

## Graphical abstract

**Figure:**
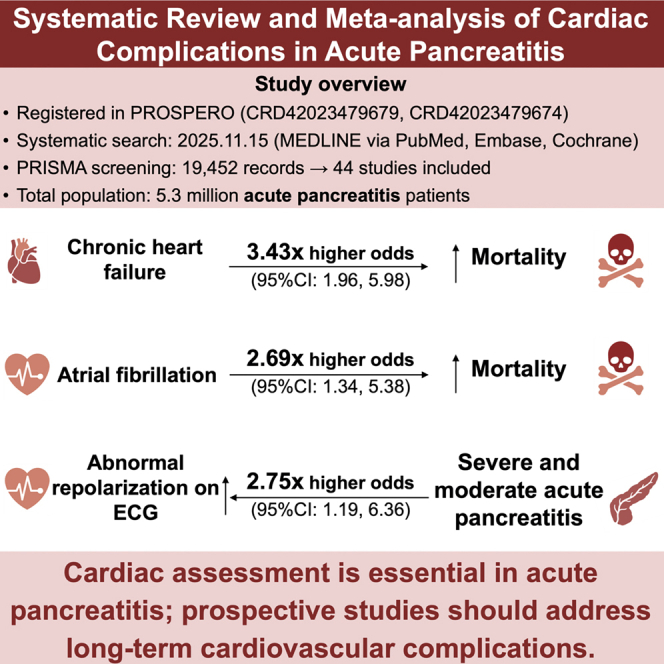

## Introduction

Acute pancreatitis (AP) is a systemic inflammatory disorder with an incidence of 33–74 cases per 100,000 person-years^1^; the main causes are alcohol consumption, gallstones, and hypertriglyceridaemia.^2^ AP is associated with systemic inflammation that can affect multiple organs, including the heart.^3^ In AP, mortality is alarmingly high, with in-hospital mortality ranging from 2% to 3% and post-discharge mortality increasing to 5.5% within the first year.^2^^,^^4^

Previous cohort studies suggested that pre-existing and new-onset cardiac abnormalities play a key role in the outcomes of AP. For example, pathological alterations of electrocardiography (ECG), echocardiographic (echo), and cardiac laboratory marker changes are associated with worse clinical outcomes in AP.^3^^,^^5^^,^^6^^,^^7^^,^^8^^,^^9^ On the other hand, AP can cause hypovolemia and electrolyte disturbances (hyperkalemia, hypomagnesemia, and hypophosphatemia) that can lead to arrhythmia, even cardiac failure.^3^^,^^10^^,^^11^

Data on the post-discharge management of AP are considerably limited. Although numerous studies have described cardiological changes that occur during the acute phase, the cardiac assessment of AP patients after discharge is poorly investigated.^3^^,^^5^^,^^6^^,^^7^^,^^8^^,^^9^^,^^12^ A recent registry analysis found that mortality in the first 90 days after discharge is almost as high as during hospitalization, with heart failure accounting for 22.5% of post-discharge deaths.^4^ Therefore, it is important to focus not only on the reduction of in-hospital mortality but also on post-discharge follow-up.

Routine cardiology screening is not a standard part of care of AP patients during hospitalization or after discharge. This gap in clinical knowledge and practice may play a role in the high rates of cardiac-related mortality in these patients. Therefore, we aim to explore the bidirectional association between cardiac changes and AP outcomes. Our hypothesis is that specific cardiac-related abnormalities are associated with higher mortality and worse severity in AP.

## Results

**Figure 1:**
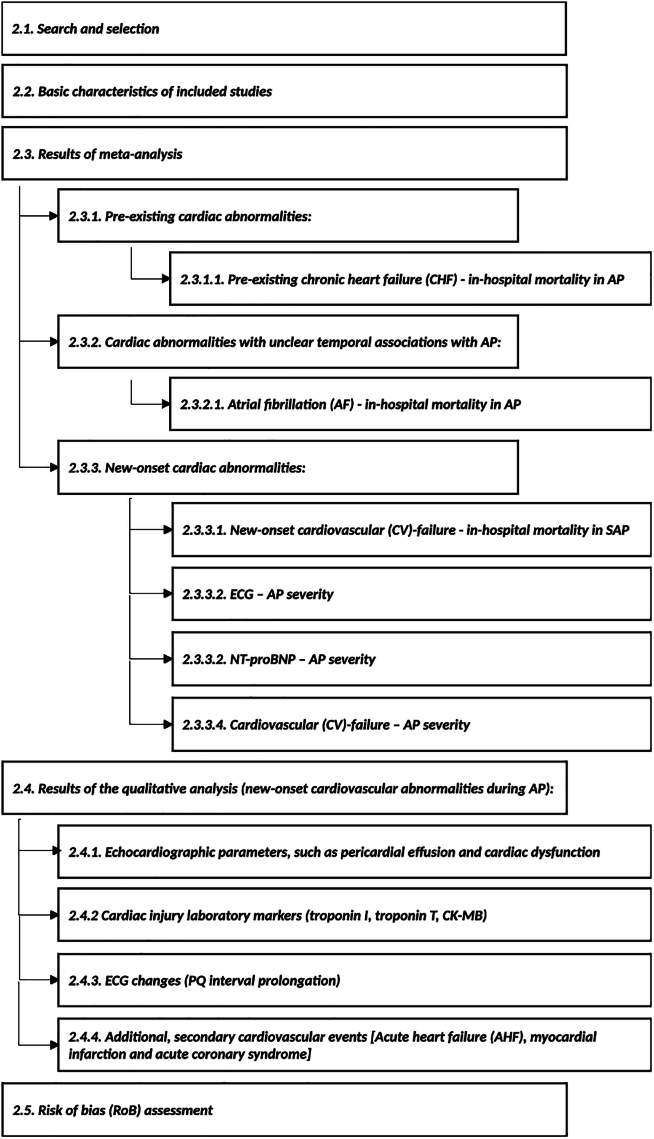
Organizational map of the results section and analyzed outcomes

To provide an overview of the analyzed outcomes and the structure of the results section, we summarized the key analysis categories and endpoints in a schematic diagram (Figure 1.).

### Search and selection

**Figure 2:**
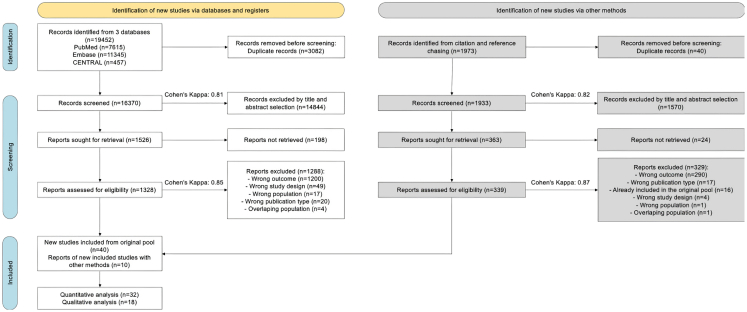
PRISMA flowchart of selection

Altogether, 19,452 studies were identified by our search key and an additional 1,973 with the backward and forward citation searching. The PRISMA flowchart of selection can be found in Figure 2. See “Data S1” supplementary file for the list of excluded full-text articles with reasons. Altogether, 42 studies were included in the meta-analysis and two articles in the systematic review.

### Basic characteristics of included studies

We included 42 cohort studies (12 prospective, 30 retrospective) and two RCTs in full-text journal article form, except for five conference abstracts and three letters to the editor with original data. Of the 5,267,772 included patients, 24.28% were female, and the mean age was 58.61 years. As several large studies drew on the US National Inpatient Sample, some degree of overlap is possible. We accounted for this by verifying or estimating study periods wherever possible (see Tables S5 and S6). Excluding all National (Nationwide) Inpatient Sample/Nationwide Readmission Database (NIS/NRD) database population (4,029,385 patients), the remaining cohort across non-database studies comprised 1,238,387 AP patients. Most of the articles included were of mixed etiologies; two of them reported on only^13^^,^^14^ hypertriglyceridemia-induced AP, one assessed^15^ alcohol-induced and two biliary AP.^16^^,^^17^ The baseline characteristics of the enrolled articles are detailed in Table 1.

**Table 1:** Baseline characteristics table

Author, year	Country	Study design	Sample size (female%)	Age (mean ± SD)	Comparisons	Outcome
**Factor: severity of AP; Outcome: cardiac abnormality**						

Abraham et al.^18^	India	RCT	129 (10)	ND	SAP vs. MAP	CV organ dysfunction
Ates et al.^16^	Turkey	prospective cohort	32 (ND)	ND	SAP vs. MAP	QTc min, max, d
Barassi et al.^19^	Italy	cohort; letter to the editor	37 (37.84)	55.80(±ND)	SAP vs. MAP	cTnT level
Chacón-Portillo et al.^8^	Mexico	prospective cohort	27 (62.96)	48 ± 17	SAP and MSAP vs. MAP	Change in ECG, echo, cardiac laboratory markers
Fan et al.^20^	Hong Kong	RCT	195 (59)	ND	SAP vs. MAP	Cardiogenic shock
						Myocardial infarction
Muller et al.^21^	Sweden	prospective cohort	109 (34.90)	56 (16–86)^c^	SAP vs. MAP	CV organ dysfunction
Nadkarni et al.^3^	India	prospective cohort	52 (15.39)	36.50 ± 11	SAP vs. MAP	QT prolongation, pericardial effusion
Park et al.^22^	Republic of Korea	retrospective cohort	672 (33.94)	SAP: 54 (47–64), MSAP+MAP: 51 (40–62)^b^	SAP vs. MSAP vs. MAP	CV failure
Pintado et al.^23^	Spain	prospective cohort	57 (28.60)	62.20 ± 15.80	SAP vs. MSAP	Cardiac arrhythmia, ACS
Podda et al.^24^	Italy	retrospective cohort	2728 (52)	53 ± 19	SAP vs. MSAP vs. MAP	CV failure, SBP, HR
Rubio-Tapia et al.^25^	Mexico	cohort	51 (41.18)	40(±ND)	SAP vs. MAP	ECG
Shi et al.^26^	China	cohort	74 (29.70)	51.43 ± 13.40	hemorrhagic and necrotic vs. edema-type AP	NT-proBNP level, LVEF, E/A, mSRs
Stimac et al.^27^	Croatia	prospective cohort; letter to the editor	303 (ND)	ND	SAP vs. MAP	Heart rate, PQ interval, ST segment depression or elevation
Thong et al.^13^	Vietnam	cross-sectional	157 (22.30)	41.50 ± 9.70	SAP vs. MSAP and MAP	Tachycardia, hypotension
Viswanathan et al.^15^	India	retrospective cohort	119 (ND)	ND	SAP vs. MSAP vs. MAP	Tachycardia, short PR, QT prolongation, HR, PR, QRS, QTc
Zhao et al.^5^	China	retrospective cohort	238 (ND)	ND	SAP vs. MSAP vs. MAP	cTNI, NT-proBNP, CK-MB

**Factor: cardiac abnormality; Outcome: clinical outcome of AP**						

Awan et al.^28^	USA	retrospective cohort	1128744 (46.50)	58.50(±ND)	ACS	Mortality, LOS
Castaneda et al.^29^	USA	retrospective cohort (NIS), conference abstract	274785 (47)	51(±ND)	AF	Mortality, ICU admission, additional LOS
Cho et al.^30^	Korea	retrospective cohort	22 (30.40)	52.10 ± 16.40	CV failure	Mortality
Fiedler et al.^31^	Germany	prospective case-control	93 (40)	ND	Shock	Mortality
Gawande et al.^32^	India	retrospective cohort	42 (11.90)	39.45 ± 11.15	Two-dimensional echocardiogram abnormalities	ICU admission
Guo et al.^33^	China	prospective cohort	447 (40)	45 (22–75)^b^	CV failure at any time during admission	Mortality
Huang et al.^34^	China	retrospective cohort; letter to the editor	136 (39.70)	ND	acute heart failure	SAP, mortality, ICU LOS
Jamal et al.^35^	USA	retrospective cohort (NIS)	575230 (46.89)	60.59(±ND)	AF	Mortality, LOS
Jiang et al.^36^	China	prospective cohort	1790 (32.35)	49^a^	elevated CK-MB	Mortality, SAP, infected pancreatic necrosis, LOS
Khan et al.^37^	USA	retrospective cohort (NIS); conference abstract	ND	ND	AF	Mortality
Khanna et al.^6^	USA	cross-sectional (NIS)	1098580 (14.20)	ND	type 2 MI	Mortality, ICU admission, additional LOS
Kroner et al.^38^	USA	retrospective cohort (NIS); conference abstract	274774 (49)	51(±ND)	CHF	Mortality
Luthra et al.^17^	USA	retrospective cohort (NRD)	97027 (62)	66.20 ± 16.70	CHF	Mortality
Maringhini et al.^9^	Italy	prospective cohort	100 (46)	54.60 ± 18.50	pericardial effusion	Mortality, SAP
Mehta et al.^39^	USA	retrospective cohort (NIS); conference abstract	1356659 (ND)	59.71(±ND)	CHF	Mortality
Mihoc et al.^40^	Romania	retrospective cohort	53 (ND)	ND	circulatory organ failure	Mortality
Nadkarni et al.^3^	India	prospective cohort	52 (15.39)	36.50 ± 11	prolonged QTc, cardiac diastolic disfunction, pericardial effusion	Mortality, surgery
Okamoto et al.^41^	Japan	retrospective cohort	115 (26.10)	53 (42–71)^b^	relative bradycardia	MSAP and SAP (CTSI, Ranson), necrosis
Prasada et al.^42^	India	retrospective cohort	65 (32.30)	39.55 ± 13.14	elevated CK-MB	Mortality, pancreatic necrosis, LOS
Q. Liu et al.^43^	USA	retrospective cohort	539 (42.10)	57 (45–71)^b^	AF, CHF	Mortality
Shah et al.^44^	USA	retrospective cohort (NIS); conference abstract	449357 (ND)	ND	AF	Mortality, LOS
Singh et al.^45^	India	retrospective cohort	50 (20)	ND	CV failure	Mortality
Spampinato et al.^7^	Italy	retrospective cohort	385 (46)	65.40 ± 18.70	CHF	Mortality
Thandassery et al.^46^	India	prospective cohort	72 (38.90)	41 (19–81)^c^	cardiac dysfunction, DID	Mortality
Tran et al.^47^	Netherlands	retrospective cohort	267 (53.90)	ND	CV failure	Mortality
Valverde-López et al.^48^	Spain	prospective cohort	227 (50.30)	65.30 ± 18.90	Rise in heart rate	SAP
Walker et al.^49^	United Kingdom	retrospective cohort	2027 (ND)	56^a^	new onset cardiac disease	ICU admission
Wu et al.^50^	China	retrospective cohort	523 (57.20)	59.20 ± 17.40	AF, heart failure, AMI	Mortality
Z. Liu et al.^14^	USA	retrospective cohort	631 (44.84)	60.38 (47.08–72.54)^b^	AF, CHF	Mortality

ACS, acute coronary syndrome; AF, atrial fibrillation; CHF, chronic heart failure; CK-MB, creatine kinase-myocardial band; criteria of Muller: Muller NL: imaging of the pleura. radiology 186:297-309, 1993; cTNI, cardiac troponin I; cTnT, cardiac troponin T; CTSI, Balthazar computed tomography severity index; CV, cardiovascular; DID, diastolic dysfunction; E/A, mitral e peak and a peak ratio; echo, echocardiography; HR, heart rate; ICU, intensive care unit; LAHB, left anterior hemiblock; LVEF, left ventricular ejection fraction; MABP, mean arterial blood pressure; MAP, mild acute pancreatitis; MI, myocardial infarction; MODS, multi organ dysfunction syndrome; MSAP, moderate acute pancreatitis; mSRs, mean of systolic strain rate peak; NIS, National (Nationwide) Inpatient Sample; NRD, Nationwide Readmission Database; NT-proBNP, N-terminal pro B-type natriuretic peptide; RBBB, right bundle branch block; RCT, randomized controlled trial; RWMA, regional wall motion abnormality; SAP, severe acute pancreatitis; SBP, systolic blood pressure; SYD, systolic dysfunction.

a median.

b median (Q1-Q3).

c median (minimum-maximum).

### Results of meta-analysis

#### Pre-existing cardiac abnormalities

##### Pre-existing chronic heart failure is associated with higher odds of in-hospital mortality in AP

**Figure 3:**
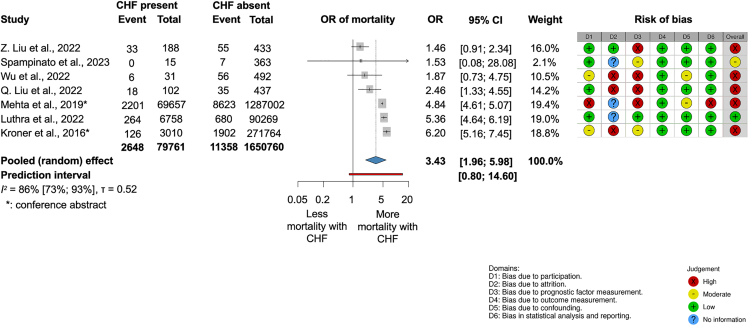
Chronic heart failure (CHF) is associated with higher odds of in-hospital mortality in acute pancreatitis (AP)

In an analysis of more than 1.7 million AP patients from 7 retrospective cohort studies, including nationwide samples (Table S5.), those with pre-existing chronic heart failure (CHF) had a 3-fold increased odds of mortality (OR [odds ratio], 3.43; confidence interval [CI], 1.96–5.98; I^2^ = 86% [73%–93%]). (Figure 3).

#### Cardiac abnormalities with unclear temporal associations with AP

##### Atrial fibrillation of unclear onset is associated with higher odds of in-hospital mortality in AP

**Figure 4:**
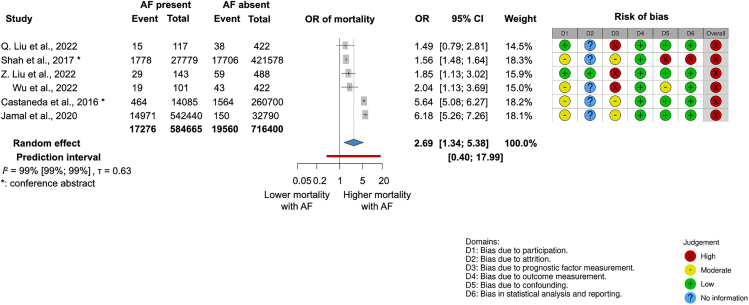
Atrial fibrillation (AF) of unclear onset is associated with higher odds of in-hospital mortality in acute pancreatitis (AP)

The analysis of 1.3 million AP patients from six retrospective cohort studies,^14^^,^^29^^,^^35^^,^^43^^,^^44^^,^^50^ including nationwide samples (Table S5), showed that those with atrial fibrillation (AF) had almost three times higher odds of in-hospital mortality than patients without AF (OR, 2.69; CI, 1.34–5.38; I^2^ = 99% [99%–99%]) (Figure 4). In these studies, the temporal relationship of AF to AP was not explicitly stated, and therefore, it is unclear whether AF was pre-existing or developed during AP.

#### New-onset cardiac abnormalities

##### New-onset CV failure is associated with higher odds of in-hospital mortality in SAP

**Figure 5:**
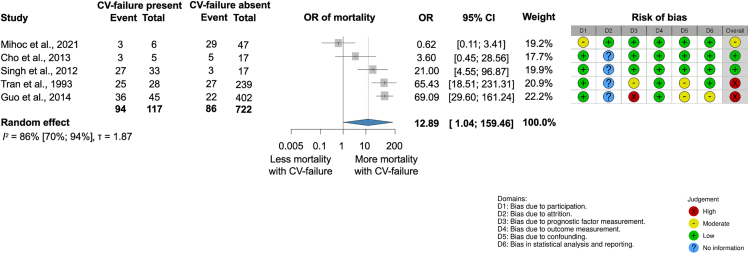
Cardiovascular failure (CV failure) is associated with higher odds of in-hospital mortality in severe acute pancreatitis (SAP)

We could identify only studies (four retrospective^30^^,^^40^^,^^45^^,^^47^ and one prospective^33^) reporting data on severe acute pancreatitis (SAP) (*n* = 839) patients for analyzing new-onset cardiovascular (CV) failure and AP mortality. Patients with new-onset CV failure had almost thirteen times higher odds of death in SAP than patients without CV failure (OR, 12.89; CI, 1.04–159.46; I^2^ = 86% [70%–94%]) (Figure 5.). The definition of CV failure in each study can be found in Table S7.

##### There are higher odds of new-onset abnormal repolarization on ECG in (M)SAP; however, we did not find sufficient evidence yet for a significant association between the severity of AP and QTc prolongation on ECG compared to MAP

**Figure 6:**
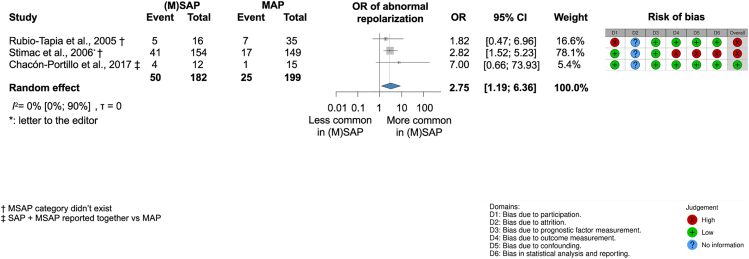
Moderate and severe acute pancreatitis ([M]SAP) is associated with higher odds of abnormal repolarization on ECG compared to mild acute pancreatitis (MAP) † MSAP category did not exist; ‡ SAP + MSAP reported together vs. MAP.

**Figure 7:**
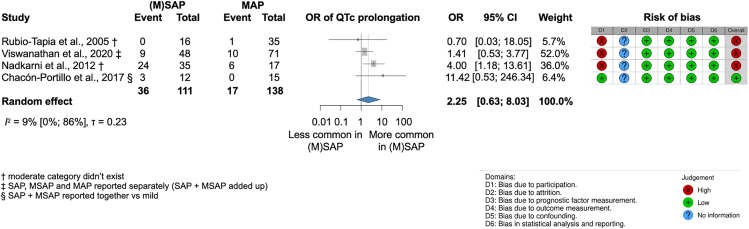
Moderate and severe acute pancreatitis ([M]SAP) is not significantly associated with higher odds of QTc prolongation on ECG compared to mild acute pancreatitis (MAP) † moderate category did not exist, ‡ SAP, MSAP, and MAP reported separately (SAP + MSAP added up), § SAP + MSAP reported together vs. MAP.

Patients with severe and moderately severe AP ([M]SAP) had almost three times higher odds for in-hospital changes in new-onset repolarization on ECG (flat ST, ST depression, elevation, T-wave inversion, and nonspecific changes) than patients in the mild (MAP) category (OR, 2.75; CI, 1.19–6.36; I^2^ = 0% [0%–90%]) analyzing 381 patients from two prospective studies (Stimac et al. 2006 and Chacón-Portillo et al. 2017)^8^^,^^27^ and one retrospective cohort study (Rubio-Tapia et al. 2005)^25^ (Figure 6). Compared to MAP, the OR for in-hospital new-onset QTc interval prolongation on ECG among (M)SAP patients was 2.25 (CI, 0.63–8.03) (I^2^ = 9% [0%–86%]) analyzing 249 patients from two prospective studies (Nadkarni et al. 2012 and Chacón-Portillo et al. 2017)^3^^,^^8^ and two retrospective studies (Rubio-Tapia et al. 2005 and Viswanathan et al. 2020)^15^^,^^25^ (Figure 7). However, we did not find sufficient evidence for a significant association yet.

##### There is no sufficient evidence yet for a significant association between the severity of AP and NT-proBNP levels

**Figure 8:**
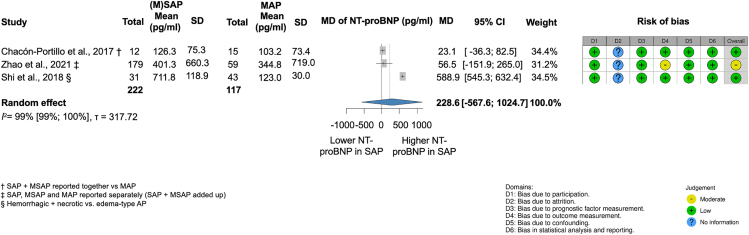
Moderate and severe acute pancreatitis ([M]SAP) is not significantly associated with higher levels of N-terminal pro-B-type natriuretic peptide (NT-proBNP; pg/ml) compared to mild acute pancreatitis (MAP) † SAP + MSAP reported together vs MAP, ‡ SAP, MSAP and MAP reported separately (SAP + MSAP added up), § Hemorrhagic + necrotic vs. edema-type AP.

Analyzing 339 patients from two retrospective (Zhao et al. 2021 and Shi et al. 2018)^5^^,^^26^ and one prospective (Chacón-Portillo et al. 2017)^8^ cohort study,^5^^,^^8^^,^^26^ we found that NT-proBNP levels in serum were not significantly higher in patients with (M)SAP compared to those with MAP (mean difference [MD], 228.6 pg/mL; CI, 567.6–1,024.7 pg/mL; I^2^ = 99% [99%–100%]) (Figure 8).

##### SAP is associated with new onset CV failure

**Figure 9:**
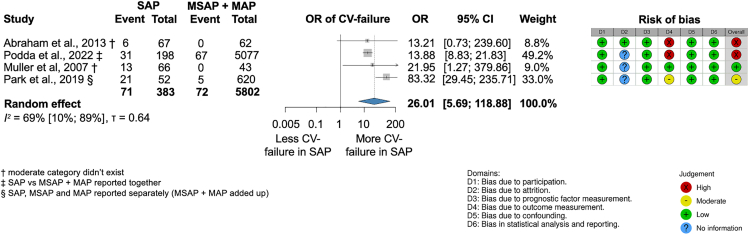
Severe acute pancreatitis (SAP) is associated with higher odds for cardiovascular failure (CV failure) compared to moderate and mild acute pancreatitis (MSAP and MAP) † moderate category did not exist, ‡ SAP vs MSAP + MAP reported together, § SAP, MSAP, and MAP reported separately (MSAP + MAP added up).

The odds of acute CV failure were higher in SAP than in MSAP and MAP (OR, 26.01; CI, 5.69–118.88; I^2^ = 69% [10%–89%]) (Figure 9.) when analyzing 3,638 patients from two prospective^18^^,^^21^ and two retrospective studies.^22^^,^^24^ The definition of CV failure in each study can be found in Table S7.

### Results of the qualitative analysis on new-onset cardiovascular abnormalities during AP

#### Echocardiographic parameters, such as pericardial effusion and cardiac dysfunction are associated with worse AP outcomes

Nadkarni et al. (OR, 2.67, CI, 0.29–24.83)^3^ and Maringhini et al. (OR, 1.71, CI, 0.31–9.30)^9^ found no significant association of pericardial effusion with mortality. However, the latter study found five times higher odds of SAP (OR, 5.0, CI, 1.50–17.0) in the presence of pericardial effusion.^9^ Thandassery et al.^46^ and Gawande et al.^32^ found that the risk of mortality and the odds of ICU admission were 22 times higher in AP with cardiac dysfunction on echo (HR [hazard ratio], 22.07, CI, 1.26–387.74 and OR, 25.6, CI, 2.36–278.12, respectively), but Nadkarni et al.^3^ found no significant associations (OR, 13.38, CI, 0.72–247.28). Furthermore, left ventricular diastolic diameter, regional wall motion abnormality, left ventricle ejection fraction , mitral e peak and a peak ratio, mean of systolic strain rate peak, hypokinesis, right ventricle dilation, and hypertrophy, pulmonary hypertension, tricuspid regurgitation, mitral regurgitation, aortic insufficiency, left atrial dilation, and pulmonary insufficiency were investigated, but none showed significant differences in the outcomes of AP.^8^^,^^25^^,^^26^

#### Cardiac injury laboratory markers are associated with worse AP outcomes

Jamal et al. 2020 and Prasada et al. 2018 found that elevated creatine kinase-myocardial band (CK-MB) levels were associated with six times higher mortality (OR, 6.18, CI, 5.26–7.26 and OR, 6.42, CI, 1.00–38.90, respectively) and longer length of stay (LOS) (MD, 1.91 days and MD, 7.00 days, CI, 1.99–12.01, respectively).^35^^,^^42^ Levels of CK-MB were lower in MAP (MD, −3.38 ng/mL, CI, −6.09 to −0.67).^5^ cTnT and cTnI levels did not significantly change in SAP (MD, 72.30 pg/mL, CI: −171.63–316.23 and MD: −0.03 ng/mL, CI: −0.08–0.01, respectively).^5^^,^^19^

#### ECG changes, such as PQ interval prolongation, are associated with more severe AP

In terms of ECG changes, only PQ interval was significantly prolonged in SAP in one study (MD: 111.30 ms, CI: 96.22–126.38)^27^; otherwise, bradycardia, signs of left atrial enlargement, short/long PR interval, QRS prolongation, inferior QS complex, left anterior hemiblock, right bundle branch block , inferior hemiblock, supraventricular premature contractions, and respiratory arrhythmia were not significantly associated with AP outcomes.^8^^,^^15^^,^^16^^,^^25^^,^^27^^,^^41^^,^^51^

#### Additional, secondary cardiovascular events associated with acute pancreatitis, like acute heart failure, myocardial infarction, and acute coronary syndrome are associated with worse AP outcomes

In this subsection, we summarize studies that investigated secondary cardiovascular events complicating AP with adverse outcomes. Huang et al.^34^ investigated abnormal acute heart failure (AHF) after fluid resuscitation in SAP patients defined by hemodynamic criteria (cardiac index <3.0, global end-diastolic volume index >700, extravascular lung water index >10) and found significant associations between AHF and the severity of AP (OR, 5.41, CI, 1.20–24.31) and longer ICU stay (MD, 8.95 days, CI, −9.36 to −8.54), although mortality was not significantly higher among AP patients with AHF (OR, 1.84, CI, 0.46–7.43). On the contrary, Wu et al.^50^ showed two times higher odds of mortality in AP when patients had AHF according to ICD codes (OR, 2.04, CI, 1.13–3.69) during the disease course. Myocardial infarction (types 1 and 2) occurring during AP was associated with higher odds of mortality (OR, 2.40, CI, 1.50–3.80; OR, 7.65, CI, 1.0579–55.3174, respectively), intensive care unit admission (OR, 3.30, CI, 2.30–4.80), and additional LOS (OR, 2.10, CI, 1.40–2.80),^6^^,^^50^ but were not significantly associated with the severity of AP (OR, 7.20, CI, 0.34–152.04).^20^ Mortality (OR, 16.90, CI, 13.50–21.30) and LOS (MD, 4.00 days) were higher in patients with acute coronary syndrome as a complication; although it was not significantly associated with the severity of AP (OR, 2.78, CI, 0.14–55.23).^23^^,^^28^

Summarized data for our systematic review can be found in Table 2.

**Table 2:** Summary of the qualitative analysis

Factor: severity of AP; Outcome: cardiac abnormality				
**Author, year**	**Comparisons**	**Outcome**	**MD**	**CI 95%**
Ates et al.^16^	SAP vs. MAP	QTc min (ms)	−5.00	−27.97–17.97
		QTc max (ms)	−10.00	−44.56–24.55
		QTcd (ms)	−5.00	−13.66–3.66
Barassi et al.^19^	SAP vs. MAP	cTnT level (pg/mL)	72.30	−171.63–316.23
Han et al.^52^	SAP vs. MSAP vs. MAP	MABP (Hgmm)	−0.40	−6.03–5.23
Shi et al.^26^	hemorrhagic and necrotic vs. edema-type AP	LVEF (%)	14.13	9.88–18.38
		E/A	0.61	0.51–0.71
		mSRs	0.31	0.22–0.40
Stimac et al.^27^	SAP vs. MAP	PQ interval (ms)	111.30	96.22–126.38
Viswanathan et al.^15^	SAP + MSAP vs. MAP	PR (ms)	−11.35	−20.68–−2.02
		QRS (ms)	1.80	−2.44–6.04
		QTc (ms)	−10.55	−22.48–1.38
Wang et al.^53^	SAP + MSAP vs. MAP	MABP (Hgmm)	4.06^a^	−1.77–9.89^a^
Zhao et al.^5^	SAP vs. MSAP vs. MAP	cTNI (ng/mL)	−0.03	−0.08–0.01
		CK-MB (ng/mL)	−3.38	−6.09–−0.67
**Author, year**	**Comparisons**	**Outcome**	**OR**	**CI 95%**
Chacón-Portillo et al.^8^	SAP and MSAP vs. MAP	Tachycardia	1.33	0.22–8.22
		Bradycardia	4.67	0.42–52.12
		QRS prolongation	4.04	0.15–108.57
		RBBB	4.04	0.15–108.57
		LAHB	7.38	0.32–169.82
		LA abnormality	4.04	0.15–108.57
		Inferior QS complex	4.04	0.15–108.57
		Right ventricle dilation	4.04	0.15–108.57
		Diastolic dysfunction	0.59	0.05–7.43
		Pulmonary hypertension	1.96	0.39–9.93
		Tricuspid regurgitation	0.80	0.11–5.77
		Mitral regurgitation	0.22	0.01–4.95
		Aortic insufficiency	0.59	0.05–7.43
		Left atrial dilation	4.04	0.15–108.57
		Hypokinesis	0.39	0.01–10.37
		Pulmonary insufficiency	1.24	0.02–67.04
		Increased TnI	2.17	0.30–15.71
		Increased Pro-BNP	0.88	0.19–4.00
Fan et al.^20^	SAP vs. MAP	Cardiogenic shock	45.13	2.64–771.34
		Myocardial infarction	7.20	0.34–152.04
Nadkarni et al.^3^	SAP vs. MAP	Pericardial effusion	2.67	0.29–24.83
Pintado et al.^23^	SAP vs. MSAP	cardiac arrythmia	1.29	0.24–6.94
		ACS	2.78	0.14–55.23
Rubio-Tapia et al.^25^	SAP vs. MAP	LAHB	0.52	0.05–5.03
		Flat ST	0.41	0.02–8.95
		Long QT	0.70	0.023–18.045
		Bradycardia	0.70	0.03–18.05
		RBBB	0.70	0.03–18.05
		Supraventricular premature contractions	0.40	0.02–8.95
		Right ventricle hypertrophy	0.70	0.03–18.05
		Respiratory arrhythmia	6.87	0.26–178.24
		Inferior hemiblock	0.70	0.03–18.05
Thong et al.^13^	SAP vs. MSAP and MAP	hypotension	10.83	3.19–36.76
Viswanathan et al.^15^	SAP + MSAP vs. MAP	Short PR	0.49	0.18–1.36

Factor: cardiac abnormality; Outcome: clinical outcome of AP				
**Author, year**	**Factor**	**Outcome**	**MD**	**CI 95%**
Awan et al.^28^	ACS	Length of stay (days)	4.00	N/A
Francisco et al.^51^	low SBP	Length of stay (days)	−1.35^a^	−2.64–−0.07^a^
	bradycardia (low HR)		−1.30^a^	−2.56–−0.04^a^
Huang et al.^34^	abnormal heart failure	ICU hospital stay (days)	8.95	−9.36–−8.54
Jamal et al.^35^	elevated CK-MB	Length of stay (days)	1.91	N/A
Prasada et al.^42^	elevated CK-MB	Length of stay (days)	7.00	1.99–12.01
Shah et al.^44^	AF	Length of stay (days)	−2.10	N/A
**Author, year**	**Factor**	**Outcome**	**OR**	**CI 95%**
Awan et al.^28^	ACS	Mortality	16.90^c^	13.50–21,30 †
Castaneda et al.^29^	AF	Mortality	1.87^c^	1.41–3.49 †
		ICU admission	2.23^c^	1.77–2.81 †
		Additional length of stay	1.50^c^	1.23–1.77 †
Cho et al.^30^	CV failure	Mortality	3.60	0.45–28.56
Fiedler et al.^31^	Shock	Mortality	5.50	1.85–16.34
Gawande et al.^32^	cardiac involvement	ICU admission	3.05	0.31–29.97
	Two-dimensional echocardiogram abnormalities	ICU admission	25.60	2.36–278.12
Guo et al.^33^	CV failure at any time during admission	mortality	69.09	29.60–161.25
Huang et al.^34^	abnormal heart failure	SAP	5.41	1.20–24.31
		Mortality	1.84	0.46–7.43
Jamal et al.^35^	elevated CK-MB	Mortality	6.18	5.26–7.26
Khan et al.^37^	AF	Mortality	1.90^b^	1.47–2.57^b^
Khanna et al.^6^	type 2 MI	Mortality	2.40^b^	1.50–3.80^b^
		ICU admission	3.30^b^	2.30–4.80^b^
		Additional length of stay	2.10^b^	1.40–2.80^b^
Maringhini et al.^9^	pericardial effusion	Mortality	1.71	0.31–9.30
		SAP	5.00	1.50–17.00
Mihoc et al.^40^	circulatory organ failure	Mortality	0.62	0.11–3.41
Nadkarni et al.^3^	prolonged QTc	Mortality	17.00	0.92–312.52
		Surgery	10.50	1.23–89.69
	cardiac diastolic disfunction	Mortality	13.38	0.72–247.28
		Surgery	2.09	0.48–9.02
	pericardial effusion	Mortality	8.20	1.29–52.16
Okamoto et al.^41^	relative bradycardia	MSAP and SAP	0.40	0.16–0.98
		SAP	0.18	0.02–1.47
		SAP	0.17	0.04–0.77
		Necrosis	0.13	0.02–1.06
Prasada et al.^42^	elevated CK-MB	Mortality	6.42	1.00–38.90
		Pancreatic necrosis	2.44	0.50–12.30
		Mortality	1.46	0.20–8.80
Q. Liu et al.^43^	AF	Mortality	1.49	0.79–2.81
Shah et al.^44^	AF	Mortality	1.56	1.48–1.64
Singh et al.^45^	CV failure	Mortality	21.00	4.55–96.87
Thandassery et al.^46^	cardiac dysfunction	Mortality	22.07^c^	1.26–387.74^c^
	DID		3.60^c^	1.10–12.50^c^
Tran et al.^47^	CV failure	Mortality	65.43	18.51–231.32
Valverde-López et al.^48^	Rise in heart rate	SAP	5.80^b^	1.29–25.98^b^
Walker et al.^49^	new onset cardiac disease	ICU admission	1.61	N/A
Wu et al.^50^	AF	Mortality	1.87	0.73–4.75
	Heart failure		2.04	1.13–3.69
	AMI		7.65	1.0579–55.3174
Z. Liu et al.^14^	AF	Mortality	1.85	1.13–3.02

MD, mean difference; CI, confidence interval; SAP, severe acute pancreatitis; MSAP, moderate acute pancreatitis; MAP, mild acute pancreatitis; QTcmin, corrected minimum QT interval; QTcmax, corrected maximum QT interval; QTcd, corrected QT dispersion; MODS, multi organ dysfunction syndrome; ACS, acute coronary syndrome; AF, atrial fibrillation; cTnT, cardiac troponin T; MI, myocardial infarction; MABP, mean arterial blood pressure; CK-MB, creatine kinase-myocardial band; ECHO, echocardiography; LAHB, left anterior hemiblock; RBBB, right bundle branch block; mSRs, mean of systolic strain rate peak; E/A, mitral e peak and a peak ratio; LVEF, left ventricular ejection fraction; SYD, Systolic dysfunction; DID, Diastolic dysfunction; cTNI, cardiac troponin I; RWMA, regional wall motion abnormality.

a estimated from median and Q1, Q3.

b adjusted.

c hazard ratio.

### Risk of bias assessment

**Figure 10:**
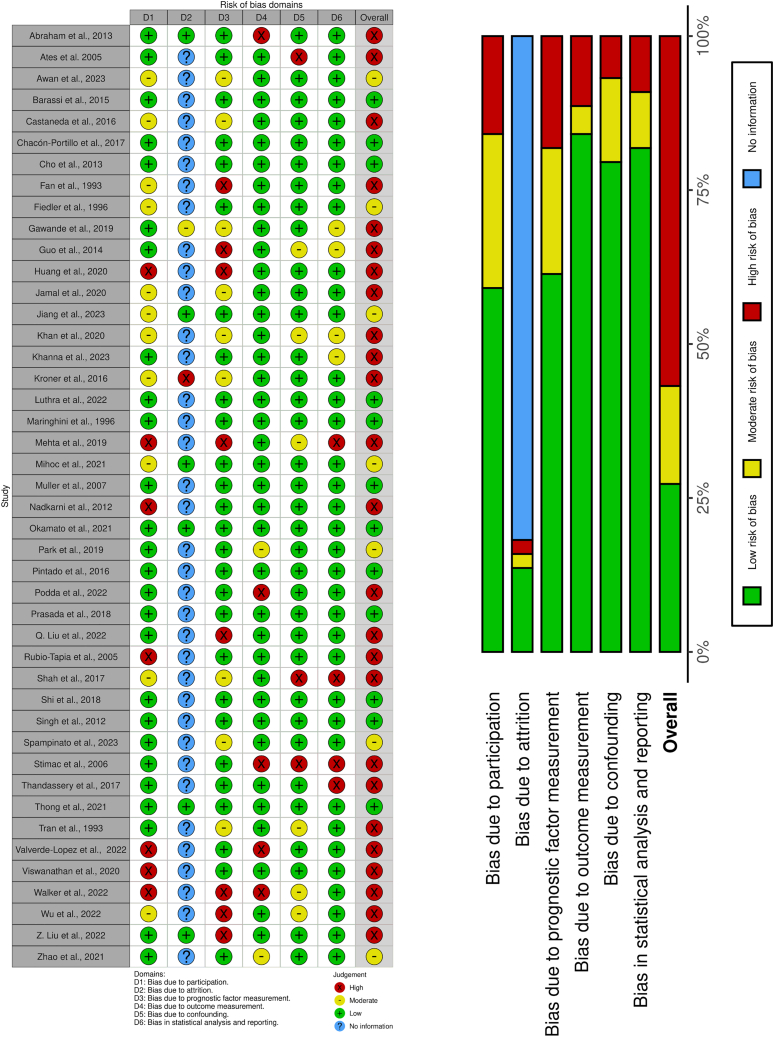
Overall risk of bias assessment using the QUIPS tool

Of the 50 articles, 28 had high, seven moderate, and 15 low risk of bias (RoB). Overall, we found a high RoB due to the retrospective, observational nature of the studies included. The results of the RoB assessment are shown in each of the outcome figures (Figures 3, 4, 5, 6, 7, 8, and 9) and the overall RoB figure on Figure 10. We found no evidence for the small study reporting bias based on forest plots, although the assessment is limited due to the small number of studies. Egger’s test could not be performed due to the low (less than ten) number of studies. Funnel plots assessing publication bias are shown in Figures S1–S7.

## Discussion

Our study found significant associations between cardiac abnormalities, such as pre-existing CHF, concurrent AF, newly developed CV failure, and the mortality of AP. Furthermore, in severe cases, there were significantly higher odds of having in-hospital cardiac failure, tachycardia, and repolarization changes on ECG.

When investigating the significance of pre-existing cardiac conditions, our findings revealed that pre-existing CHF, characterized by structural or functional heart abnormalities with a 50% five-year mortality rate, significantly increases AP-related mortality.^54^^,^^55^ Most of the included studies defined CHF based on ICD-9 and ICD-10 codes (International Statistical Classification of Diseases and Related Health Problems by WHO), or they did not give a proper definition.^14^^,^^17^^,^^38^^,^^39^^,^^43^^,^^50^ The aggressive fluid therapy commonly used in AP may exacerbate CHF through volume overload,^12^ while chronic hypoxia and hypoperfusion due to reduced cardiac output may amplify the systemic inflammatory response syndrome (SIRS) triggered by AP.^50^^,^^54^ Furthermore, a recent mechanistic work (Smichi et al., 2025) showed that patients with CHF are associated with a 1.57-times higher in-hospital mortality if they have AP. Their experimental models demonstrated that pancreatic injury can aggravate heart failure through excessive fatty acid release driven by pancreatic lipase-mediated fat necrosis, while pharmacologic inhibition or genetic deletion of pancreatic lipases prevents this deterioration.^56^

Atrial fibrillation is a supraventricular tachyarrhythmia that leads to uncoordinated atrial electrical activation and ineffective contraction; roughly one in three persons is at a lifetime risk.^57^ The odds of mortality in AF is 1.5–1.9 times higher compared to the general population.^58^ Potential triggers of AF during AP include fluid resuscitation, causing volume overload leading to atrial myocardial stretch,^59^ and autonomic imbalance between sympathetic and parasympathetic nervous systems leading to heart-rate variability.^59^^,^^60^ AF is a significant prognostic factor for morbidity and mortality due to the risks of thromboembolism, stroke, and congestive heart failure.^61^ This high-prevalence disease should be taken into consideration during AP because we found that concurrent AF increased the odds of mortality remarkably by 2.7 times.

Pre-existing and concurrent cardiac diseases, particularly CHF and AF, are well-established risk factors that exacerbate outcomes across various acute medical conditions, including COVID-19^62^^,^^63^ and pneumonia.^64^^,^^65^ While these associations are significant and cardiac diseases universally elevate the risk of mortality of any condition, their role in gastrointestinal diseases, such as AP, gastrointestinal bleeding, and diverticulitis, is underinvestigated. The interplay between cardiac comorbidities and AP imposes a severe clinical burden, potentiating patient vulnerability through the combined effects of systemic inflammation, volume shifts, and organ crosstalk, similar to what has been described in other systemic diseases such as COVID-19 or pneumonia.

Myocardial damage during AP arises from SIRS, oxidative stress, metabolic disturbances, and circulatory complications like hypovolemia during shock. These factors can exacerbate pre-existing cardiac conditions or induce new dysfunctions.^3^^,^^10^^,^^11^^,^^66^^,^^67^^,^^68^^,^^69^ While the myocardial depressant factor or proteolytic pancreatic enzymes have been implicated as triggering factors, current evidence suggests myocardial injury is largely secondary to SIRS, potentially through coronary artery spasms.^66^^,^^67^ The low peripheral resistance and myocardial depression caused by SIRS result in hemodynamic changes in AP. Numerous scores in AP, such as APACHE II, BISAP, and Modified Marshall Organ Failure Score, already include CV failure and other hemodynamic parameters, such as HR and blood pressure^70^^,^^71^^,^^72^ to predict severity and mortality. CV failure was defined according to the criteria used in the included studies, which varied across publications. Definitions were based on the Marshall Organ Failure Score,^30^^,^^40^ the revised Atlanta Classification, the American College of Chest Physicians/Society of Critical Care Medicine (1991),^45^ or study-specific thresholds (e.g., mean arterial pressure below 50 mmHg, systemic blood pressure above 100 mmHg requiring vasoactive support, or HR below 50 beats/min).^47^ We did not impose a single definition but pooled the data according to the criteria reported by each study (see Table S7). Our study further confirmed that the presence of CV failure is strongly associated with worse AP outcomes. Acute myocardial damage can lead to myocardium-specific enzyme increase, e.g., elevated CK-MB, NT-proBNP, cTnI, and cTnT levels; however, associations with AP outcomes could not be established due to limited data.^5^^,^^8^^,^^19^

ECG changes during AP can be observed for multiple causes, such as myocardial damage and metabolic and electrolyte disturbances.^73^^,^^74^ Repolarization changes (e.g., ST elevation or depression and T-wave inversion) can indicate myocardial injury and hypocalcaemia caused by pancreatic necrosis,^73^ while QTc prolongation may result from hypocalcemia, hypokalemia, hypomagnesemia or medications like antibiotics and ondansetron. QTc prolongation is particularly concerning due to its association with ventricular arrhythmias and post-discharge mortality.^3^^,^^10^^,^^68^^,^^69^^,^^75^ Hypophosphatemia can decrease myocardial contractility and may lead to rhabdomyolysis.^3^^,^^10^^,^^76^ Our analysis demonstrated significant associations between ECG repolarization changes and AP severity, but further investigation is needed to clarify other ECG changes, including QTc prolongation.

Echo findings such as reduced left ventricular stroke volume, regional wall motion abnormalities, and pericardial effusion also highlight the cardiac impact of AP.^77^ Pericardial effusions are frequent in AP; however, they are not fully understood, possibly resulting from hematogenous transfer of pancreatic enzymes or pericardial fat necrosis.^3^^,^^9^^,^^78^ Moreover, communication between the pancreatic region and the pericardial area has been demonstrated.^78^ While our meta-analysis could not evaluate echocardiographic parameters due to data limitations, qualitative evidence suggests these abnormalities warrant further study.

Post-discharge cardiac complications remain underexplored due to insufficient follow-up data. Yet, studies highlight substantial cardiac-related mortality after AP. For example, cardiac failure accounted for 22.5% of deaths within 90 days of discharge,^4^ while myocardial infarction was responsible for 38.5% of post-discharge deaths over seven years.^79^ This excess cardiac mortality may be explained by the fact that the myocardial damage during AP may occur undetected and can deteriorate further over time.

### Strengths of the study

The strength of our study is that this is the first comprehensive analysis of cardiac abnormalities in AP, for which we pre-published our methodology protocol. The high heterogeneity of the AF, CV failure, and mortality analyses is due to the fact that the population included all the severity categories, which gives us a broader, more generalizable picture of the patients, which strengthens our results.

### Implications for practice

Implementing scientific findings in clinical practice is crucial.^80^^,^^81^ Screening for cardiac comorbidities should be an essential part of clinical practice on admission in AP. Our findings suggest that early cardiac assessment should be performed in patients with existing cardiac conditions at the onset of AP to identify and manage potential exacerbations or complications. In those with cardiac comorbidities or at high cardiovascular risk, comprehensive cardiac monitoring should be performed throughout the course of AP, including ECG, echocardiography, and laboratory tests to assess cardiac function.

### Implications for research

Our results indicate that the focus should be on understanding the bidirectional interaction between the heart and the pancreas during AP, where dysfunction in one organ may worsen the other. High-quality, prospective clinical studies should be prioritized to explore this interaction. In addition, given the elevated cardiac-related mortality following AP, research should extend beyond the acute phase to include follow-up periods, ensuring a better understanding of and response to long-term cardiovascular complications after discharge.

Patients with pre-existing CHF have a 3.43 fold, and those with concurrent AF a 2.69-fold increased odds of mortality in AP, while severe and moderately severe cases show a 2.75 times higher likelihood of ECG repolarization abnormalities.

### Limitations of the study

In terms of the limitations of this study, although we could include studies with large patient populations, the number of articles for each analysis is relatively low. We included mostly retrospective observational studies, which led to imprecise definitions of certain cardiac factors and time frames in the studies. For example, the papers did not consistently specify AF onset and CHF stages and did not state the cause of death or what could lead to the worse mortality. As a result, we were unable to determine whether death was due to thromboembolism, bleeding, uncontrolled arrhythmia, or other complications. Furthermore, we included studies assessing the NIS database, where study periods were not always explicitly reported, and some degree of overlap may exist. We provided the reported study periods in the supplementary material (Tables S5 and S6). This could also inflate the total number of patients reported (5.3 million); therefore, we recalculated the aggregate, excluding all NIS/NRD studies, which still amounted to approximately 1.2 million patients. This indicates that our conclusions are robust even when large administrative datasets are omitted. Additionally, we also included conference abstracts that lessen the publication bias but contain more unreliable data. Moreover, different severity scores were used in the articles. A further limitation may be that there is an interobserver bias in certain cardiac tests, such as echo and ECG evaluation. The observed overall high risk of bias of the studies is another limitation.

## Resource availability

### Lead contact

Further information and requests for resources should be directed to and will be fulfilled by the lead contact, Dr. Rita Nagy (nagy.rita3@semmelweis.hu).

### Materials availability

This study did not generate new unique reagents.

### Data and code availability

The datasets analyzed during this study are publicly available from the original publications included in the meta-analysis. No new code was generated in this study.

## Acknowledgments

No ethical approval was required for this systematic review with meta-analysis, as all data were already published in peer-reviewed journals. No patients were involved in the design, conduct or interpretation of our study. This study was supported by 10.13039/501100002332Semmelweis University Research and Innovation Fund. Funding was provided by the Centre for Translational Medicine, Semmelweis University. Furthermore, funding was provided by the EKÖP-2024-11 and EKÖP-2024-224, University Research Scholarship Program from the source of the 10.13039/501100018818National Research, Development, and Innovation Fund Hungary (for R.N. and V.L., respectively). Sponsors had no role in the design, data collection, analysis, interpretation, and manuscript preparation.

## Author contributions

V.L. (guarantor of the article): conceptualization, project administration, methodology, data curation, visualization, and writing – original draft; M.O.: conceptualization, methodology, project administration, and writing – review & editing; D.S.V.: conceptualization, formal analysis, and writing – review & editing; P.F.: conceptualization and writing – review & editing; E.B.: conceptualization, data curation, and writing – review & editing; A.P.: conceptualization, data curation, and writing – review & editing; A.M.: conceptualization, data curation, and writing – review & editing; P.H.: conceptualization, supervision, and writing – review & editing; R.N.: conceptualization, supervision, and writing – review & editing. All authors certify that they have participated sufficiently in the work to take public responsibility for the content, including participation in the concept, design, analysis, writing, or revision of the manuscript.

## Declaration of interests

The authors declare no competing interests.

## STAR★Methods

### Key resources table

**Table:** 

REAGENT or RESOURCE	SOURCE	IDENTIFIER
**Deposited data**		

MEDLINE (via PubMed)	National Library of Medicine	https://pubmed.ncbi.nlm.nih.gov
Embase	Elsevier	https://www.embase.com
Cochrane CENTRAL	Cochrane Library	https://www.cochranelibrary.com
PROSPERO	University of York^82^	CRD42023479679, CRD42023479674

**Software and algorithms**		

R (version 4.3.2)	R Foundation for Statistical Computing^83^	https://www.r-project.org
meta R package	Guido Schwarzer^84^	https://cran.r-project.org/package=meta
dmetar R package	David D. Ebert^85^	https://dmetar.protectlab.org
robvis tool	McGuinness & Higgins^86^	https://mcguinness.shinyapps.io/robvis
EndNote X9	Clarivate Analytics	https://endnote.com
Microsoft Excel (Office 365)	Microsoft	https://www.microsoft.com
PRISMA2020 ShinyApp	Haddaway et al.^87^^,^^88^	https://www.eshackathon.org/software/PRISMA2020.html
citationchaser (R package)	Haddaway et al.^89^	https://github.com/ropensci/citationchaser

**Other**		

QUIPS risk of bias tool	Hayden et al.^90^	https://methods.cochrane.org/prognosis/quips-tool

### Experimental model and subject details

This study is a systematic review and meta-analysis and does not involve experimental models, animals, or human subjects.

### Method details

#### Study design and registration

We conducted a systematic review and meta-analysis following the Preferred Reporting Items for Systematic Reviews and Meta-Analyses (PRISMA) 2020 guideline^87^ (Table S1), the Cochrane Handbook^91^ and the Meta-analysis Of Observational Studies in Epidemiology (MOOSE) group^92^ (Table S2). The study protocols were registered on PROSPERO for two question frameworks (CRD42023479679 and CRD42023479674).^82^ The only minor deviation from the protocols was including studies that did not exclude patients with pre-existing cardiac conditions. An analysis of pre-existing chronic heart failure (CHF) was also performed.

#### Eligibility criteria

We applied the PFO (patients, factors, outcomes) framework to define eligibility.^93^ Randomized controlled trials (RCTs), observational studies, and case series containing at least ten patients reporting on (P) patients with AP regardless of age were included. The diagnosis of AP requires two of the following three criteria: characteristic abdominal pain, elevated serum amylase or lipase levels (to at least three times the normal upper limit), and distinct findings of AP on imaging.^72^ The first framework focused on patients (P) with AP and cardiac abnormalities (F). Outcomes (O) included mortality, severity of AP, and length of hospital stay (LOS). AP severity was classified using the revised Atlanta criteria^72^ into mild (MAP), moderately severe (MSAP), and severe (SAP), based on the presence and duration of local complications and organ failure. Before 2012, there were only two categories: severe and non-severe AP. The second framework examined AP severity as the factor (F) and cardiac abnormalities as outcomes (O). The following cardiac abnormalities were investigated as factors in the first framework: CV-failure, ECG changes like sinus tachycardia/bradycardia, atrial fibrillation, left atrial enlargement signs, short/long PR/PQ interval, QRS prolongation, inferior QS complex, abnormal repolarization (flat ST, ST elevation, ST depression, T-wave inversion, non-specific changes), QTc interval prolongation, left anterior hemiblock (LAHB), right bundle branch block (RBBB), inferior hemiblock, supraventricular premature contractions and respiratory arrhythmia. Furthermore, we investigated ECHO changes like systolic dysfunction (SYD), diastolic dysfunction (DID), pericardial effusion, left ventricular diastolic diameter (LVDD), regional wall motion abnormality (RWMA), right ventricle dilation, pulmonary hypertension, tricuspid regurgitation, mitral regurgitation, aortic insufficiency, left atrial dilation, hypokinesis, pulmonary insufficiency, left ventricle ejection fraction (LVEF), peak velocity blood flow from left ventricular relaxation in early diastole to peak velocity flow in late diastole caused by atrial contraction (E/A), mean of systolic strain rate peak (mSRs) and heart rate variability. Moreover, we researched cardiac laboratory markers changes like cTnI, cTnT, NT-proBNP and CK-MB.

The exclusion criteria were animals, malignancy, and preexisting severe cardiac diseases (e.g., heart failure, previous myocardial infarct).

#### Information sources and search strategy

We systematically searched MEDLINE (via PubMed), Embase, and Cochrane CENTRAL on November 5, 2023. A backward and forward citation search was conducted on February 19, 2024 using a reference-checking tool.^89^ No language or other restrictions were applied. Our search key included two main domains focusing on AP and cardiac-related terms. For a detailed search key, see Table S3.

#### Study selection

After the systematic search, the resulting articles were managed using EndNote, and duplicates were removed. During the selection, two independent reviewers (VL and AP, EB, AM) screened titles, abstracts, and full texts, with a third reviewer (MO) resolving disagreements. Cohen’s kappa coefficient (κ) assessed interrater agreement. Missing full text files were requested from the authors via email. Table S4 provides examples of studies that might appear to meet the inclusion criteria but were excluded.

#### Data collection and variables extracted

Data were manually extracted into an Excel spreadsheet (Office 365, Microsoft, Redmond, WA, USA) by two reviewers (VL and AP, EB, AM), with disagreements resolved by a third (MO). Extracted data included first author, year of publication, countries, study design, main study findings, patient demographics (age and sex), factors and outcomes with time-frames and definitions (cardiac-related abnormalities such as ECG, echocardiographic, cardiac laboratory marker abnormalities; and hospital/post-discharge mortality, severity of AP, length of hospital stay).

#### Risk of bias assessment

Two independent authors (VL and AP, EB, AM) assessed the methodological quality of each study using the Quality In Prognosis Studies (QUIPS) tool^90^ as recommended by the Cochrane Handbook.^91^ A third reviewer resolved potential disagreements (MO).

### Quantification and statistical analysis

Both a qualitative and quantitative synthesis of the data was performed. The minimum number of studies to perform a meta-analysis was three.

As we assumed considerable between-study heterogeneity in all cases, a random-effects model was applied to pool effect sizes using frequentist methods. Odds ratio (OR) was used as an effect size measure for binary outcomes. To calculate the OR and the pooled odds ratio, the total number of patients and those with the event of interest in each group separately was extracted from the studies. Where only adjusted OR was given, we used it in a separate analysis. The difference between the mean (MD) was used for the effect size measure of continuous outcomes. Sample size, mean and corresponding standard deviation (SD) were extracted or estimated from each study (in each group separately) to calculate the study MDs and pooled MDs. We reported the results as the odds of event of interest in the “exposed” group versus the odds of event of interest in the “control” group, or the mean in the “exposed” group minus the mean in the “control” group. The exposure in the first framework was the presence of a cardiac abnormality, the control group was patients without the cardiac abnormality. We also made comparisons based on the severity of the disease and investigated which abnormalities were more likely to be present in certain severity groups. Pooled OR based on the total number of patients and events of interest in each group was calculated using the Mantel-Haenszel method. The inverse variance weighting method was used to calculate the pooled OR (based on OR directly given without total and event number) and MD.

Results were considered statistically significant if the pooled 95% confidence interval (CI) did not contain the null value. We summarized the findings of the meta-analysis in forest plots. Between-study heterogeneity was described by the between-study variance (*τ*^2^) and Higgins and Thompson’s *I*^2^ statistics.^94^ We reported the prediction interval when the study number was above 5. Small study publication bias was assessed by visual inspection of funnel-plots and calculating Egger (for continuous) or Harbord (for dichotomous) test p-value^95^ when at least ten studies were involved. Potential outlier publications were explored using different influence measures and plots as recommended by Harrer et al.^85^ If at least three studies with low or moderate ROB were available, a subset analysis of those studies was conducted. All statistical analyses were calculated by R software^83^ using the *meta* package^84^ for basic meta-analysis calculations and plots, and *dmetar* package^96^ for additional influential analysis calculations and plots.

Additional figures were created by PRISMA2020,^88^ PowerPoint (Office 365, Microsoft, Redmond, WA, USA) and Risk-of-bias VISualization (robvis) tools.^86^ Additional details on the statistical analysis can be found in the supplemental information (Methods S1 for the Methods).

## Supplemental information

Document S1. Figures S1–S7, Tables S1–S6, and Method S1

Data S1. List of excluded full-text articles with reasons
