# Supplementary material for: Systematic review and meta-analysis of cardiac complications in acute pancreatitis
Source: iScience. 2025 Dec 4;29(1):114172. doi: 10.1016/j.isci.2025.114172 (PMC12804618; doi:10.1016/j.isci.2025.114172)
Supplement: Document S1. Figures S1–S7, Tables S1–S6, and Method S1 [file mmc1.pdf]

iScience, Volume *29*

Supplemental information

Systematic review and meta-analysis of cardiac complications in acute pancreatitis

Veronika Lillik, Mahmoud Obeidat, Dániel Sándor Veres, Péter Ferdinandy, Elizabet Bodó, Asal Pourrastegar, Ali Moradi, Péter Hegyi, and Rita Nagy

SUPPLEMENTAL INFORMATION

METHODS S1. SUPPLEMENTAL INFORMATION FOR THE METHODS

Additional information on synthesis methods

The exact Mantel-Haenszel method (without continuity correction) was used to handle zero cell counts (as recommended by Cooper and Sweeting [1, 2]). To estimate the tau-square for OR calculation, the Paule-Mandel method was used for the other outcome measures (restricted), and the maximum likelihood estimator was used with the Q profile method for a confidence interval. We used a Hartung-Knapp adjustment [3, 4] for CIs. This adjustment was applied only if it is more conservative then the classical one (as recommended in Jackson et al. [5] as hybrid method 2).

If the study reported median, lower and upper quartiles instead of the mean and SD, we used the Luo and Shi methods [6, 7] (as implemented in the used meta R package [8]) to estimate the mean and SD. We assumed that the distribution is not relevantly differ form a lognormal distribution regarding this estimation. As the lower and upper quartiles were relatively symmetrical to the median where it was given, and most of the studies reported the mean and SD, we think this assumption is acceptable.

If the study reported separately the mean and SD for independent groups e.g. as severe and moderate severe AP, we combined it using the following equations:

$$M_{combined} = \frac{N_1 * M_1 + N_2 * M_2}{N_1 + N_2} \text{ and}$$

$$SD_{combined} = \sqrt{\frac{(N_1-1)*SD_1^2+(N_2-1)*SD_2^2+\frac{N_1*N_2}{N_1+N_2}*(M_1^2+M_2^2-2*M_1*M_2)}{N_1*N_2-1}}$$

Where Ms are the means, Ns are the sample sizes and SDs are the standard deviations of the corresponding situation (combined, group1, group2).

In the article Yihui Wang et al., 2021 [9], we combined the groups based on mean and SD after the mean and SD were estimated from the quartiles.

For pooling the effect size, pooled OR was calculated by the Mantel-Haenszel method [10, 11]. Exact Mantel-Haenszel method (without continuity correction) was used to handle zero cell counts.

On the forest plots, t-distribution based method used for CI of MD calculation of individual studies. For OR, normal approximation CI was shown on the forest plot. In case of 0 cell counts, individual study OR and CI was calculated by adding 0.5 as continuity correction (it was used only for visualization on forest plot).

Potential outlier publications were explored using different influence measures and plots – although, as the study number was small, it has limited diagnostic value and we did not reported it in our publication.

SUPPLEMENTAL FIGURE AND TABLE LEGENDS:

Table S1. PRISMA2020 checklist

Table S2. MOOSE Checklist for Meta-analyses of Observational Studies

Table S3. The detailed search key

Table S4. Studies that might appear to meet the inclusion criteria but were excluded

Table S5. Study periods of NIS/NRD studies for chronic heart failure and mortality outcomes (Fig. 3)

Table S6. Study periods of NIS/NRD studies for atrial fibrillation and mortality outcomes (Fig. 4)

Figure S1. Funnel-plot for the analysis of comorbid chronic heart failure and mortality in acute pancreatitis (Fig. 3.)

Figure S2. Funnel-plot for the analysis of atrial fibrillation and in-hospital mortality in acute pancreatitis (Fig. 4.).

Figure S3. Funnel-plot for the analysis of cardiovascular failure and mortality in acute pancreatitis (Fig. 5.)

Figure S4. Funnel-plot for the analysis of abnormal repolarization on ECG and severity of acute pancreatitis (Fig. 6.).

Figure S5. Funnel-plot for the analysis of QTc prolongation on ECG and severity of acute pancreatitis (Fig. 7.)

Figure S6. Funnel-plot for the analysis of N-terminal pro–B-type natriuretic peptide (NT-proBNP) and severity of acute pancreatitis (Fig. 8.)

Figure S7. Funnel-plot for the analysis of cardiovascular failure and severity of acute pancreatitis (Fig. 9.).

Table S1. PRISMA2020 checklist

Section and Topic	Item #	Checklist item	Location where item is reported
**TITLE**			
Title	1	Identify the report as a systematic review.	Page 1
**ABSTRACT**			
Abstract	2	See the PRISMA 2020 for Abstracts checklist.	Page 3
**INTRODUCTION**			
Rationale	3	Describe the rationale for the review in the context of existing knowledge.	Page 5
Objectives	4	Provide an explicit statement of the objective(s) or question(s) the review addresses.	Page 5
**METHODS**			
Eligibility criteria	5	Specify the inclusion and exclusion criteria for the review and how studies were grouped for the syntheses.	Page 31
Information sources	6	Specify all databases, registers, websites, organisations, reference lists and other sources searched or consulted to identify studies. Specify the date when each source was last searched or consulted.	Page 33
Search strategy	7	Present the full search strategies for all databases, registers and websites, including any filters and limits used.	Suppl. Mat. Table 2
Selection process	8	Specify the methods used to decide whether a study met the inclusion criteria of the review, including how many reviewers screened each record and each report retrieved, whether they worked independently, and if applicable, details of automation tools used in the process.	Page 33
Data collection process	9	Specify the methods used to collect data from reports, including how many reviewers collected data from each report, whether they worked independently, any processes for obtaining or confirming data from study investigators, and if applicable, details of automation tools used in the process.	Page 33
Data items	10a	List and define all outcomes for which data were sought. Specify whether all results that were compatible with each outcome domain in each study were sought (e.g. for all measures, time points, analyses), and if not, the methods used to decide which results to collect.	Page 33
	10b	List and define all other variables for which data were sought (e.g. participant and intervention characteristics, funding sources).	Page 33

Section and Topic	Item #	Checklist item	Location where item is reported
		Describe any assumptions made about any missing or unclear information.	
Study risk of bias assessment	11	Specify the methods used to assess risk of bias in the included studies, including details of the tool(s) used, how many reviewers assessed each study and whether they worked independently, and if applicable, details of automation tools used in the process.	Page 34-35
Effect measures	12	Specify for each outcome the effect measure(s) (e.g. risk ratio, mean difference) used in the synthesis or presentation of results.	Page 34-35
Synthesis methods	13a	Describe the processes used to decide which studies were eligible for each synthesis (e.g. tabulating the study intervention characteristics and comparing against the planned groups for each synthesis (item #5)).	Page 34-35
	13b	Describe any methods required to prepare the data for presentation or synthesis, such as handling of missing summary statistics, or data conversions.	Page 34-35
	13c	Describe any methods used to tabulate or visually display results of individual studies and syntheses.	Page 34-35
	13d	Describe any methods used to synthesize results and provide a rationale for the choice(s). If meta-analysis was performed, describe the model(s), method(s) to identify the presence and extent of statistical heterogeneity, and software package(s) used.	Page 34-35 and Supple. Mat. Page 3-4
	13e	Describe any methods used to explore possible causes of heterogeneity among study results (e.g. subgroup analysis, meta-regression).	Page 34-35
	13f	Describe any sensitivity analyses conducted to assess robustness of the synthesized results.	NA
Reporting bias assessment	14	Describe any methods used to assess risk of bias due to missing results in a synthesis (arising from reporting biases).	Page 35, Supplementary Figures S1-7
Certainty assessment	15	Describe any methods used to assess certainty (or confidence) in the body of evidence for an outcome.	NA
**RESULTS**			
Study selection	16a	Describe the results of the search and selection process, from the number of records identified in the search to the number of studies included in the review, ideally using a flow diagram.	Page 7, Fig. 2
	16b	Cite studies that might appear to meet the	Suppl. Table

Section and Topic	Item #	Checklist item	Location where item is reported
		inclusion criteria, but which were excluded, and explain why they were excluded.	S4
Study characteristics	17	Cite each included study and present its characteristics.	Table 1.
Risk of bias in studies	18	Present assessments of risk of bias for each included study.	Page 14, Figure 3-10
Results of individual studies	19	For all outcomes, present, for each study: (a) summary statistics for each group (where appropriate) and (b) an effect estimate and its precision (e.g. confidence/credible interval), ideally using structured tables or plots.	Figure 3-10
Results of syntheses	20a	For each synthesis, briefly summarise the characteristics and risk of bias among contributing studies.	Page 7-12
	20b	Present results of all statistical syntheses conducted. If meta-analysis was done, present for each the summary estimate and its precision (e.g. confidence/credible interval) and measures of statistical heterogeneity. If comparing groups, describe the direction of the effect.	Page 7-12
	20c	Present results of all investigations of possible causes of heterogeneity among study results.	Page 7-12
	20d	Present results of all sensitivity analyses conducted to assess the robustness of the synthesized results.	NA
Reporting biases	21	Present assessments of risk of bias due to missing results (arising from reporting biases) for each synthesis assessed.	14, Supplementary Figures S1-7
Certainty of evidence	22	Present assessments of certainty (or confidence) in the body of evidence for each outcome assessed.	NA
**DISCUSSION**			
Discussion	23a	Provide a general interpretation of the results in the context of other evidence.	Page 16-19
	23b	Discuss any limitations of the evidence included in the review.	Page 19
	23c	Discuss any limitations of the review processes used.	Page 19
	23d	Discuss implications of the results for practice, policy, and future research.	Page 19-20
**OTHER INFORMATION**			
Registration and protocol	24a	Provide registration information for the review, including register name and registration number, or state that the review was not registered.	Page 31

Section and Topic	Item #	Checklist item	Location where item is reported
	24b	Indicate where the review protocol can be accessed, or state that a protocol was not prepared.	Page 31
	24c	Describe and explain any amendments to information provided at registration or in the protocol.	Page 31
Support	25	Describe sources of financial or non-financial support for the review, and the role of the funders or sponsors in the review.	Page 22
Competing interests	26	Declare any competing interests of review authors.	Page 21
Availability of data, code and other materials	27	Report which of the following are publicly available and where they can be found: template data collection forms; data extracted from included studies; data used for all analyses; analytic code; any other materials used in the review.	Page 31

From: Page MJ, McKenzie JE, Bossuyt PM, Boutron I, Hoffmann TC, Mulrow CD, et al. The PRISMA 2020 statement: an updated guideline for reporting systematic reviews. BMJ 2021;372:n71. doi: 10.1136/bmj.n71

NA: nonapplicable

Table S2. MOOSE Checklist for Meta-analyses of Observational Studies

Item No	Recommendation	Reported on Page No
**Reporting of background should include**		
1	Problem definition	5
2	Hypothesis statement	5
3	Description of study outcome(s)	31-33
4	Type of exposure or intervention used	31-33
5	Type of study designs used	31-33
6	Study population	31-33
**Reporting of search strategy should include**		
7	Qualifications of searchers (eg, librarians and investigators)	31-33
8	Search strategy, including time period included in the synthesis and key words	31-33, Supplementary Table S3
9	Effort to include all available studies, including contact with authors	31-33
10	Databases and registries searched	31-33
11	Search software used, name and version, including special features used (eg, explosion)	31-33
12	Use of hand searching (eg, reference lists of obtained articles)	31-33
13	List of citations located and those excluded, including justification	Figure 2, Supplementary Table S4
14	Method of addressing articles published in languages other than English	31-33
15	Method of handling abstracts and unpublished studies	31-33
16	Description of any contact with authors	31-33
**Reporting of methods should include**		
17	Description of relevance or appropriateness of studies assembled for assessing the hypothesis to be tested	31-33
18	Rationale for the selection and coding of data (eg, sound clinical principles or convenience)	31-33
19	Documentation of how data were classified and coded (eg, multiple raters, blinding and interrater reliability)	31-33
20	Assessment of confounding (eg, comparability of cases and controls in studies where appropriate)	31-33
21	Assessment of study quality, including blinding of quality assessors, stratification or regression on possible predictors of study results	31-33
22	Assessment of heterogeneity	31-35

23	Description of statistical methods (eg, complete description of fixed or random effects models, justification of whether the chosen models account for predictors of study results, dose-response models, or cumulative meta-analysis) in sufficient detail to be replicated	34-35, Supplementary material
24	Provision of appropriate tables and graphics	Table1-3. Supplementary Tables S3-6.
**Reporting of results should include**		
25	Graphic summarizing individual study estimates and overall estimate	Figures 3-9
26	Table giving descriptive information for each study included	Table 1
27	Results of sensitivity testing (eg, subgroup analysis)	NA
28	Indication of statistical uncertainty of findings	NA
**Reporting of discussion should include**		
29	Quantitative assessment of bias (eg, publication bias)	16-19, 34-35, Figure 3-10, Supplementary Figures S1-7
30	Justification for exclusion (eg, exclusion of non-English language citations)	Figure 2., Suppl. Tabl S4.
31	Assessment of quality of included studies	
**Reporting of conclusions should include**		
32	Consideration of alternative explanations for observed results	16-19
33	Generalization of the conclusions (ie, appropriate for the data presented and within the domain of the literature review)	16-20
34	Guidelines for future research	19-20
35	Disclosure of funding source	22

From: Stroup DF, Berlin JA, Morton SC, et al, for the Meta-analysis Of Observational Studies in Epidemiology (MOOSE) Group. Meta-analysis of Observational Studies in Epidemiology. A Proposal for Reporting. *JAMA*. 2000;283(15):2008-2012. doi: 10.1001/jama.283.15.2008.

NA: nonapplicable

Table S3. The detailed search key

Pubmed:	((acute or biliary or necrotizing) and pancreatitis) AND (cardia* or myocard* or NYHA or infarction or ecg or electrocardiogra* or long QT or QT dispersion or QT interval or QT prolongation or depolarization or repolarization or T-wave or ST or hemiblock or bundle branch block or arrhythmia or tachycardia or bradycardia or CK-MB or NT-proBNP or troponin or cTnI or cTnT or echocardiography or ventric* or valv* or systolic dysfunction or diastolic dysfunction or combined dysfunction or pericard* or ejection fraction or global longitudinal strain or contractility)
EMBASE:	(acute OR biliary OR necrotizing) AND ('pancreatitis'/exp OR pancreatitis) AND (cardia* OR myocard* OR 'nyha'/exp OR nyha OR 'infarction'/exp OR infarction OR 'ecg'/exp OR ecg OR electrocardiogra* OR 'long qt' OR (long AND qt) OR 'qt dispersion'/exp OR 'qt dispersion' OR (qt AND ('dispersion'/exp OR dispersion)) OR 'qt interval'/exp OR 'qt interval' OR (qt AND interval) OR 'qt prolongation'/exp OR 'qt prolongation' OR (qt AND prolongation) OR 'depolarization'/exp OR depolarization OR 'repolarization'/exp OR repolarization OR 't wave'/exp OR 't wave' OR 'st'/exp OR st OR 'hemiblock'/exp OR hemiblock OR 'bundle branch block'/exp OR 'bundle branch block' OR (bundle AND ('branch'/exp OR branch) AND block) OR 'arrhythmia'/exp OR arrhythmia OR 'tachycardia'/exp OR tachycardia OR 'bradycardia'/exp OR bradycardia OR 'ck mb' OR 'nt probnp' OR 'troponin'/exp OR troponin OR ctni OR ctnt OR 'echocardiography'/exp OR echocardiography OR ventric* OR valv* OR 'systolic dysfunction'/exp OR 'systolic dysfunction' OR (systolic AND dysfunction) OR 'diastolic dysfunction'/exp OR 'diastolic dysfunction' OR (diastolic AND dysfunction) OR 'combined dysfunction' OR (combined AND dysfunction) OR pericard* OR 'ejection fraction'/exp OR 'ejection fraction' OR (ejection AND fraction) OR 'global longitudinal strain'/exp OR 'global longitudinal strain' OR (('global'/exp OR global) AND ('longitudinal'/exp OR longitudinal) AND ('strain'/exp OR strain)) OR contractility)
COHRANE:	((acute or biliary or necrotizing) and pancreatitis) AND (cardia* or myocard* or NYHA or infarction or ecg or electrocardiogra* or long QT or QT dispersion or QT interval or QT prolongation or depolarization or repolarization or T-wave or ST or hemiblock or bundle branch block or arrhythmia or tachycardia or bradycardia or CK-MB or NT-proBNP or troponin or cTnI or cTnT or echocardiography or ventric* or valv* or systolic dysfunction or diastolic dysfunction or combined dysfunction or pericard* or ejection fraction or global longitudinal strain or contractility)

No filter, no additional settings in the data bases

Table S4. Studies that might appear to meet the inclusion criteria but were excluded

Author, year	Title	Reason of exclusion
Buğdacı et al. 2014 [12]	QT interval changes and reversibility of QT dispersion in patients with acute pancreatitis	QT changes of acute pancreatitis subjects during attack and after remission of the disease
Chung et al. 2017 [13]	Incidence and risk of acute coronary syndrome in patients with acute pancreatitis: A nationwide cohort study	The control group consisted of healthy individuals
Hedjoudje et al. 2021 [14]	Serum phosphate is associated with mortality among patients admitted to ICU for acute pancreatitis	Cardiac factor (non-invasive blood pressure) given as a continuous measure in survival and non-survival patients, so we could not make categories from the data
Huang et al. 2012 [15]	Electrocardiographic, Cardiac Enzymes, and Magnesium in Patients With Severe Acute Pancreatitis	Descriptive study giving ECG and cardiac enzyme abnormality data on the whole cohort, not giving severity categories or the mortality of patients within each abnormality
Ito et al. 1980 [16]	The myocardial depressant factor (MDF) in acute hemorrhagic pancreatitis	The control group consisted of healthy individuals
Jang et al. 2022 [17]	Risk of cardiovascular disease and mortality in patients with diabetes and acute pancreatitis history: a nationwide cohort study	Population is patients not only with acute pancreatitis but diabetes mellitus too
Sökmen et al. 2011 [18]	Electrocardiographic changes and importance of repolarization changes in cases with acute pancreatitis	Descriptive study giving ECG parameters on the whole cohort, not giving severity categories or the mortality of patients within each ECG abnormality
Variyam et al. 1987 [19]	Pericardial Effusion and Left Ventricular Function in Patients With Acute Alcoholic Pancreatitis	The control group consisted of healthy individuals
Wilkman et al. 2013 [20]	Early Hemodynamic Variables and Outcome in Severe Acute Pancreatitis	Cardiac factor (blood pressure, heart rate) given as a continuous measure in survival and non-survival patients, so we could not make categories from the data
Yuan et al. 2020 [21]	Acute pancreatitis concomitant with diabetic ketoacidosis: a cohort from South China	Cardiac factors (hypertonia, coronary artery disease, troponin, NT-proBNP) given, but the outcome was the presence of diabetic ketoacidosis

ECG: electrocardiography, NT-proBNP: N-terminal pro–B-type natriuretic peptide

Table S5. Study periods of NIS/NRD studies for chronic heart failure and mortality outcomes (Fig. 3)

Author, year	Study type	Country	Study period start	Study period end
Kroner et al. 2016[22]	retrospective cohort (NIS)	USA	n.d.	n.d.
Wu et al. 2022 [23]	retrospective cohort	China	n.d.	n.d.
Q. Liu et al. 2022 [24]	retrospective cohort study	USA	2008	2019
Z. Liu et al. 2022 [25]	retrospective cohort study	USA	2001	2012
Luthra et al. 2022 [26]	retrospective cohort (NRD)	USA	2010	2014
Mehta et al. 2019 [27]	retrospective cohort (NIS)	USA	2010	2014
Spampinato et al. 2023 [28]	retrospective cohort	Italy	2018	2021

NIS: National Inpatient Sample; NDR: Nationwide Readmission Database; USA: United States of America; n.d.: no data

Table S6. Study periods of NIS/NRD studies for atrial fibrillation and mortality outcomes (Fig. 4)

Author, year	Study type	Country	Study period start	Study period end
Z. Liu et al. 2022 [25]	retrospective cohort study	USA	2001	2012
Jamal et al. 2020 [29]	retrospective cohort (NIS)	USA	2016	2017
Khan et al. 2020[30]	retrospective cohort (NIS)	USA	n.d.	n.d.
Castaneda et al. 2016 [31]	retrospective cohort (NIS)	USA	n.d.	n.d.
Shah et al. 2017 [32]	retrospective cohort (MIMIC-IV Database)	USA	2010	2014
Wu et al. 2022 [23]	retrospective cohort	China	n.d.	n.d.
Q. Liu et al. 2022 [24]	retrospective cohort study	USA	2008	2019

NIS: National Inpatient Sample; MIMIC-IV: Medical Information Mart for Intensive Care IV; USA: United States of America; n.d.: no data

Table S7. CV-failure definition in the included studies

Study	Definition
**Factor: CV-failure, Outcome: mortality (Figure 5)**	
Mihoc et al. 2021 [33]	Marshal score
Cho et al. 2013 [34]	Marshal score
Singh et al. 2012 [35]	Atlanta classification and American College of Chest Physicians/Society of Critical Care Medicine in 1991
Tran et al. 1993 [36]	Mean arterial pressure ≤50mmHg. Need for volume loading and/or vasoactive drugs to maintain symbolic blood pressure above 100mmHg. Heart rate ≤50b/min. Ventricular tachycardia/fibrillation. Cardiac arrest. Acute myocardial infarction.
Guo et al. 2014 [37]	n.d.
**Factor: Severity, Outcome: CV-failure (Figure 9)**	
Abraham et al. 2013 [38]	n.d.
Muller et al. 2007 [39]	need of catecholamines to maintain the blood pressure
Park et al. 2019 [40]	n.d.
Podda et al. 2022 [41]	n.d.

CV-failure: cardiovascular failure; n.d.: no data

FIGURES:

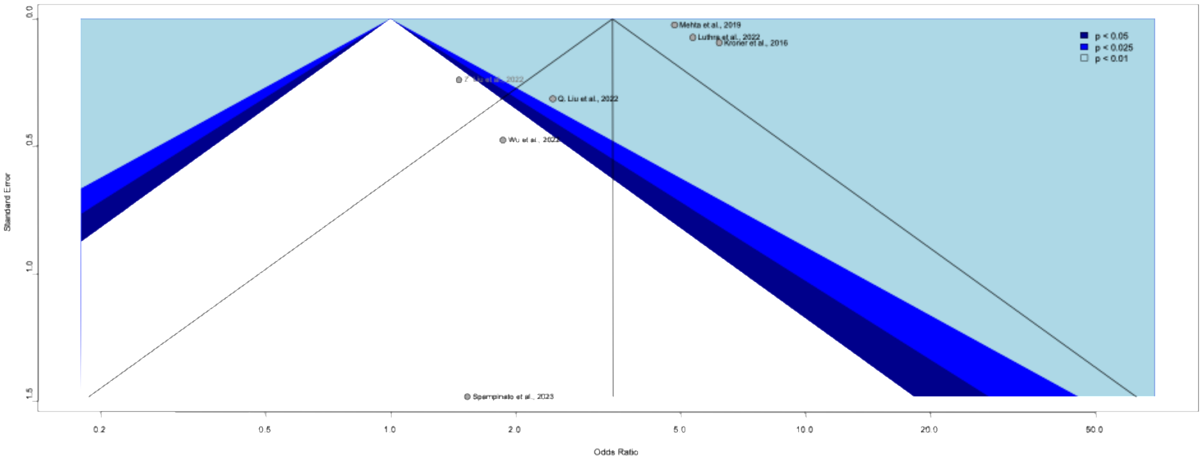

Figure S1. Funnel-plot for the analysis of comorbid chronic heart failure and mortality in acute pancreatitis (Fig. 3.)

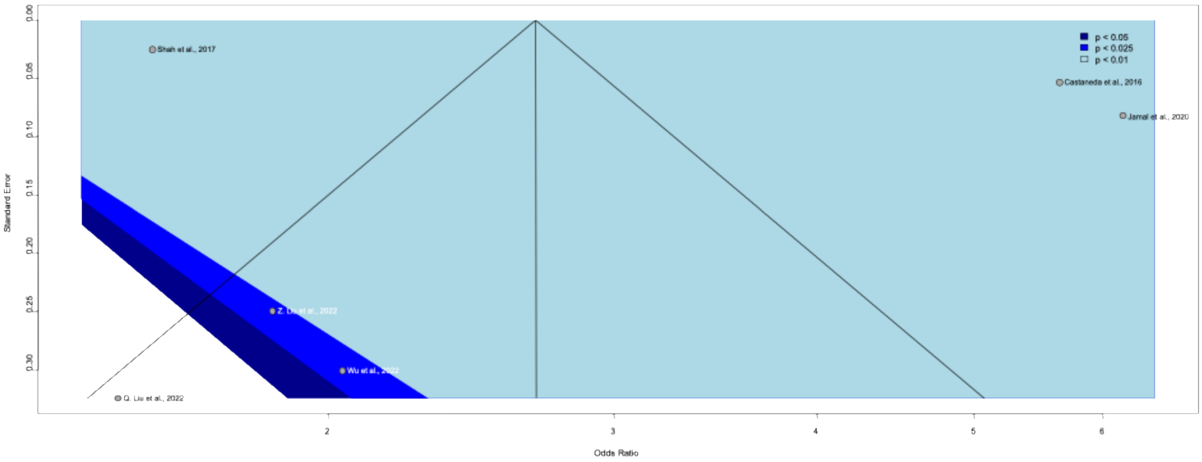

Figure S2. Funnel-plot for the analysis of atrial fibrillation and in-hospital mortality in acute pancreatitis (Fig. 4.).

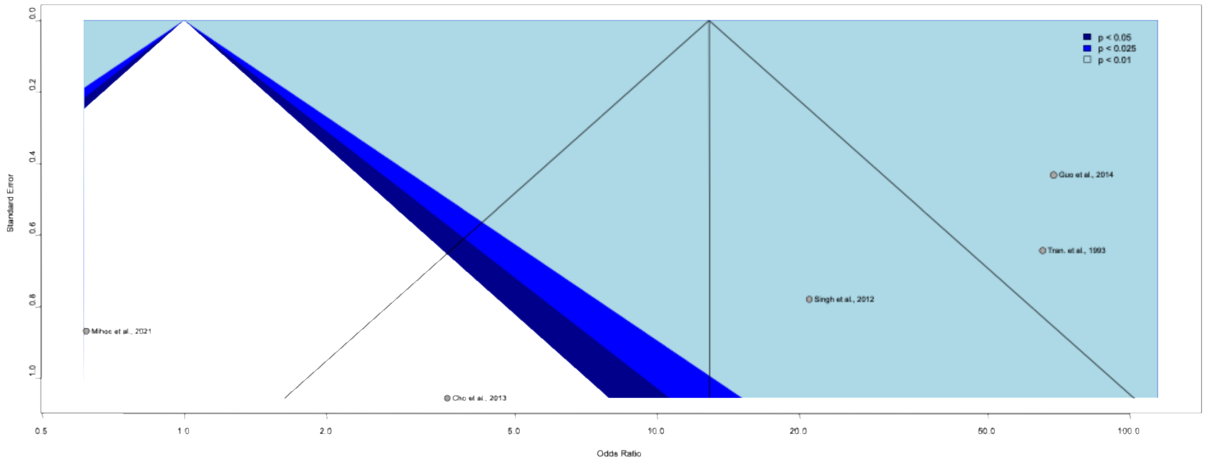

Figure S3. Funnel-plot for the analysis of cardiovascular failure and mortality in acute pancreatitis (Fig. 5.)

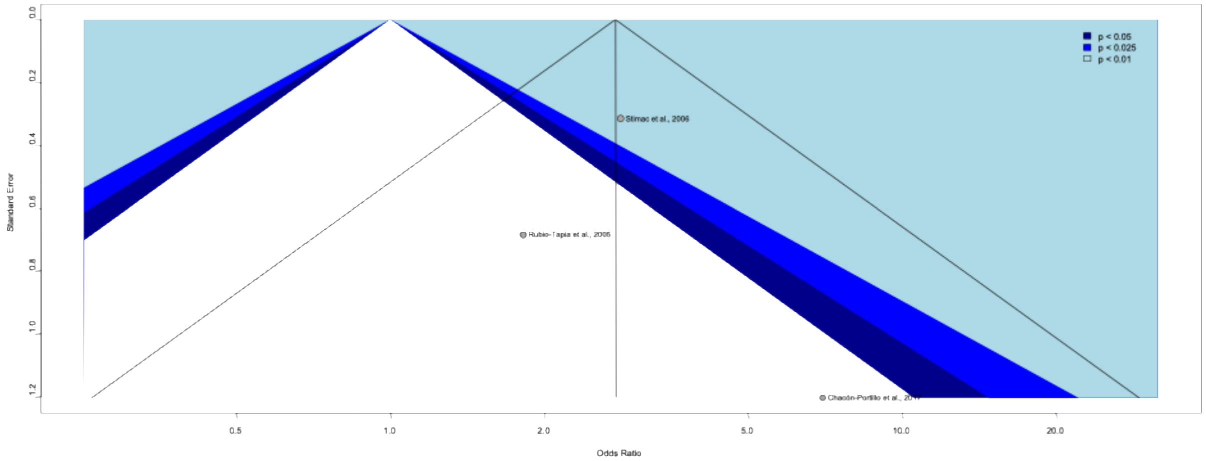

Figure S4. Funnel-plot for the analysis of abnormal repolarization on ECG and severity of acute pancreatitis (Fig. 6.).

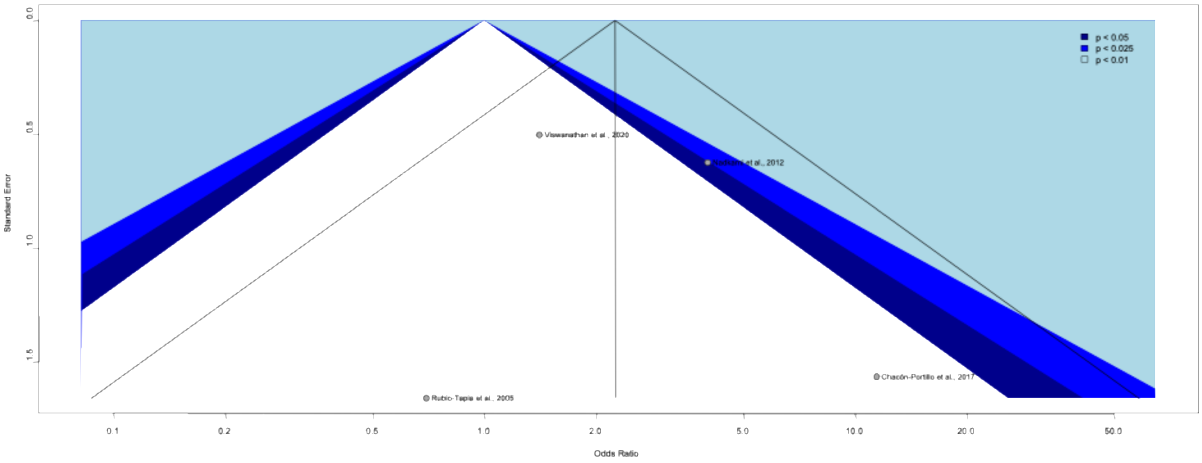

Figure S5. Funnel-plot for the analysis of QTc prolongation on ECG and severity of acute pancreatitis (Fig. 7.)

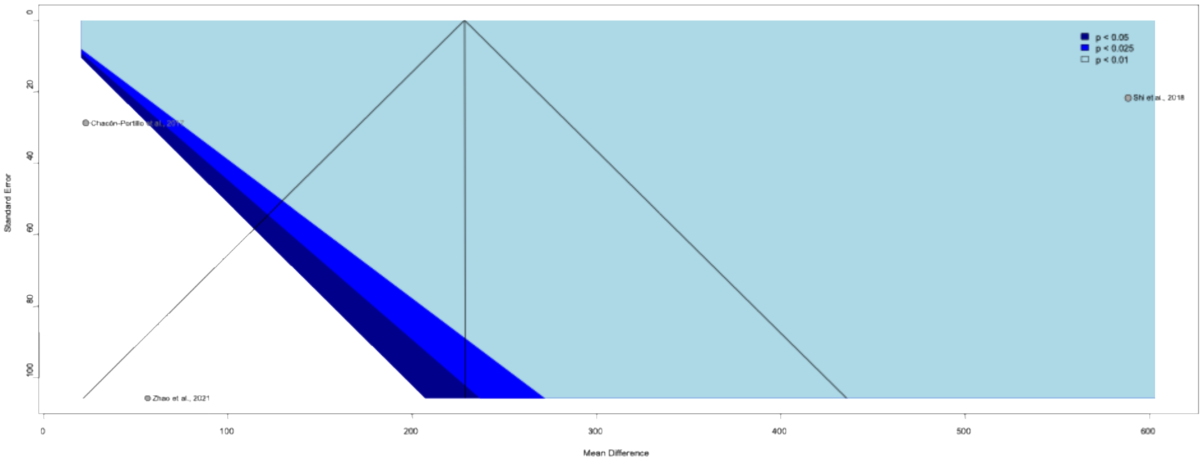

Figure S6. Funnel-plot for the analysis of N-terminal pro–B-type natriuretic peptide (NT-proBNP) and severity of acute pancreatitis (Fig. 8.)

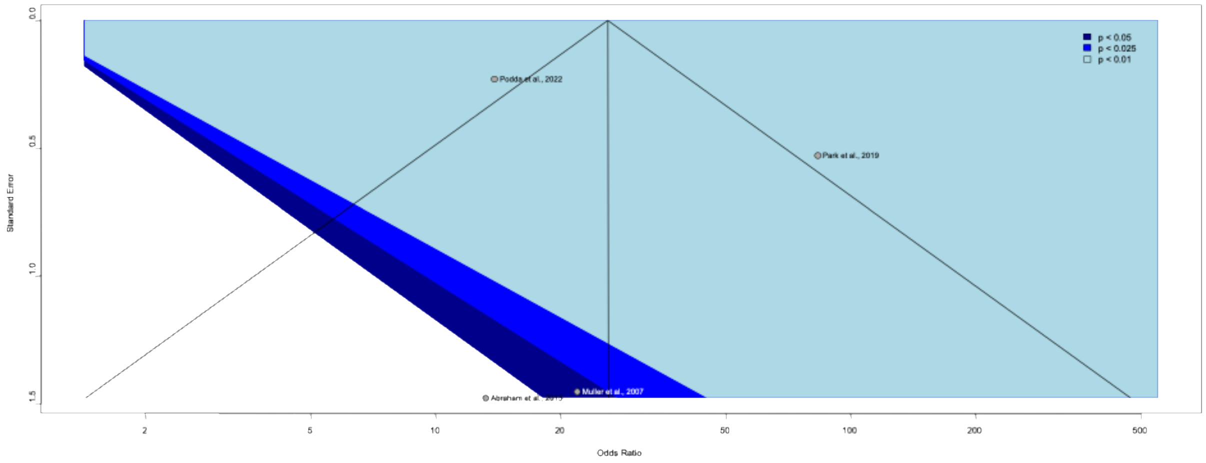

Figure S7. Funnel-plot for the analysis of cardiovascular failure and severity of acute pancreatitis (Fig. 9.).

REFERENCES:

1. Cooper H, Hedges LV and Valentine JC. *The handbook of research synthesis and meta-analysis 2nd edition*. 2009, p.1-615.

2. J. Sweeting M, J. Sutton A and C. Lambert P. What to add to nothing? Use and avoidance of continuity corrections in meta-analysis of sparse data. *Statistics in Medicine* 2004; 23: 1351-1375. DOI: https://doi.org/10.1002/sim.1761.

3. Knapp G and Hartung J. Improved tests for a random effects meta-regression with a single covariate. *Statistics in Medicine* 2003; 22: 2693-2710. DOI: https://doi.org/10.1002/sim.1482.

4. IntHout J, Ioannidis JPA and Borm GF. The Hartung-Knapp-Sidik-Jonkman method for random effects meta-analysis is straightforward and considerably outperforms the standard DerSimonian-Laird method. *BMC Medical Research Methodology* 2014; 14: 25. DOI: 10.1186/1471-2288-14-25.

5. Jackson D, Law M, Rücker G, et al. The Hartung-Knapp modification for random-effects meta-analysis: A useful refinement but are there any residual concerns? *Statistics in Medicine* 2017; 36: 3923-3934. DOI: https://doi.org/10.1002/sim.7411.

6. Luo D, Wan X, Liu J, et al. Optimally estimating the sample mean from the sample size, median, mid-range, and/or mid-quartile range. *Statistical Methods in Medical Research* 2016; 27: 1785-1805. DOI: 10.1177/0962280216669183.

7. Shi J, Luo D, Weng H, et al. Optimally estimating the sample standard deviation from the five-number summary. *Research Synthesis Methods* 2020; 11: 641-654. DOI: https://doi.org/10.1002/jrsm.1429.

8. R RCT. A language and environment for statistical computing. Vienna, Austria: R Foundation for Statistical Computing https://www.R-project.org/. 2024.

9. Wang Y, Xu Z, Zhou YH, et al. Leukocyte cell population data from the blood cell analyzer as a predictive marker for severity of acute pancreatitis. *Journal of clinical laboratory analysis* 2021; 35: e23863-NA. DOI: 10.1002/jcla.23863.

10. Mantel N and Haenszel W. Statistical Aspects of the Analysis of Data From Retrospective Studies of Disease. *JNCI: Journal of the National Cancer Institute* 1959; 22: 719-748. DOI: 10.1093/jnci/22.4.719.

11. Robins J, Greenland S and Breslow NE. A general estimator for the variance of the Mantel-Haenszel odds ratio. *Am J Epidemiol* 1986; 124: 719-723. DOI: 10.1093/oxfordjournals.aje.a114447.

12. Buğdacı MS, Tüzün A, Koca H, et al. QT interval changes and reversibility of QT dispersion in patients with acute pancreatitis. *Turk J Gastroenterol* 2014; 25 Suppl 1: 59-62. DOI: 10.5152/tjg.2014.4960.

13. Chung WS and Lin CL. Incidence and risk of acute coronary syndrome in patients with acute pancreatitis: A nationwide cohort study. *Pancreatology* 2017; 17: 675-680. 20170725. DOI: 10.1016/j.pan.2017.07.189.

14. Hedjoudje A, Farha J, Cheurfa C, et al. Serum phosphate is associated with mortality among patients admitted to ICU for acute pancreatitis. *United European Gastroenterol J* 2021; 9: 534-542. 20210505. DOI: 10.1002/ueg2.12059.

15. Huang L, Ma B, He F, et al. Electrocardiographic, cardiac enzymes, and magnesium in patients with severe acute pancreatitis. *Gastroenterol Nurs* 2012; 35: 256-260. DOI: 10.1097/SGA.0b013e31826092a6.

16. Ito K, Ramirez-Schon G, Shah PM, et al. The myocardial depressant factor (MDF) in acute hemorrhagic pancreatitis. *Trans Am Soc Artif Intern Organs* 1980; 26: 149-152.

17. Jang DK, Choi JH, Paik WH, et al. Risk of cardiovascular disease and mortality in patients with diabetes and acute pancreatitis history: a nationwide cohort study. *Sci Rep* 2022; 12: 18730. 20221104. DOI: 10.1038/s41598-022-21852-7.

18. Sökmen M, Buğdaci MS and Oztekın E. Electrocardiographic changes and importance of repolarization changes in cases with acute pancreatitis. *Turk J Gastroenterol* 2011; 22: 315-320. DOI: 10.4318/tjg.2011.0218.

19. Variyam EP and Shah A. Pericardial effusion and left ventricular function in patients with acute alcoholic pancreatitis. *Arch Intern Med* 1987; 147: 923-925.

20. Wilkman E, Kaukonen KM, Pettilä V, et al. Early hemodynamic variables and outcome in severe acute pancreatitis: a retrospective single-center cohort study. *Pancreas* 2013; 42: 272-278. DOI: 10.1097/MPA.0b013e318264c9f7.

21. Yuan S, Liao J, Cai R, et al. Acute pancreatitis concomitant with diabetic ketoacidosis: a cohort from South China. *J Int Med Res* 2020; 48: 300060520912128. DOI: 10.1177/0300060520912128.

22. Kroner PT, Castaneda D, Rojas-DeLeon M, et al. Heart Failure with Reduced Ejection Fraction Is Associated with Worse Outcomes in Patients Hospitalized with Acute Pancreatitis: Results of a Nationwide Analysis: 974. *Official journal of the American College of Gastroenterology | ACG* 2016; 111.

23. Wu M, Shi L, Zhang H, et al. Predictive value of arterial blood lactic acid concentration on the risk of all-cause death within 28 days of admission in patients with severe acute pancreatitis. *Postgraduate Medicine* 2022; 134: 210-216. Article. DOI: 10.1080/00325481.2022.2027189.

24. Liu Q, Zheng HL, Wu MM, et al. Association between lactate-to-albumin ratio and 28-days all-cause mortality in patients with acute pancreatitis: A retrospective analysis of the

MIMIC-IV database. *Frontiers in Immunology* 2022; 13. Article. DOI: 10.3389/fimmu.2022.1076121.

25. Liu Z, Yang Y, Song H, et al. A prediction model with measured sentiment scores for the risk of in-hospital mortality in acute pancreatitis: a retrospective cohort study. *Annals of Translational Medicine* 2022; 10. Article. DOI: 10.21037/atm-22-1613.

26. Luthra AK, Porter K, Hinton A, et al. A Comparison of Machine Learning Methods and Conventional Logistic Regression for the Prediction of In-Hospital Mortality in Acute Biliary Pancreatitis. *Pancreas* 2022; 51: 1292-1299. DOI: 10.1097/mpa.0000000000002208.

27. Mehta H, Shah I, Pahuja M, et al. Outcomes of Acute Pancreatitis in Patients with Heart Failure: Insights from the Nationwide Inpatient Sample. *Journal of Cardiac Failure* 2019; 25: S57-S58. DOI: 10.1016/j.cardfail.2019.07.161.

28. Spampinato MD, Caputo F, Guarino M, et al. Predicting in-hospital mortality in patients with acute pancreatitis in the ED: a direct, retrospective comparison of four clinical and radiological prognostic scores. *Minerva Gastroenterol (Torino)* 2024; 70: 147-157. 20230518. DOI: 10.23736/s2724-5985.23.03389-2.

29. Jamal S, Khan MZ, Kichloo A, et al. The Effect of Atrial Fibrillation on Inpatient Outcomes of Patients with Acute Pancreatitis: A Two-year National Inpatient Sample Database Study. *J Innov Card Rhythm Manag* 2020; 11: 4338-4344. DOI: 10.19102/icrm.2020.111205.

30. Khan MZ, Jamal S, Asim K, et al. Inpatient outcomes of acute pancreatitis with co-morbid atrial fibrillation: A cross-sectional analyses. *American Journal of Gastroenterology* 2020; 115: S1624. DOI: 10.14309/01.ajg.0000714368.91899.64.

31. Castaneda D, Kroner PT, Lemor A, et al. Atrial fibrillation is associated with worse overall outcomes in patients with acute pancreatitis-a nationwide outcome analysis. *American Journal of Gastroenterology* 2016; 111: S406-S407. DOI: 10.1038/ajg.2016.360.

32. Shah Y, Doshi S, Desai J, et al. In-hospital outcomes after concomitant occurrence of atrial fibrillation with acute pancreatitis: Insights from national inpatient sample. *American Journal of Gastroenterology* 2017; 112: S42. DOI: 10.1038/ajg.2017.295.

33. Mihoc T, Tarta C, Duta C, et al. Monitoring approach of fatality risk factors for patients with severe acute pancreatitis admitted to the intensive care unit. A retrospective, monocentric study. *Diagnostics* 2021; 11. DOI: 10.3390/diagnostics11112013.

34. Cho YS, Kim HK, Jang EC, et al. Usefulness of the Bedside Index for severity in acute pancreatitis in the early prediction of severity and mortality in acute pancreatitis. *Pancreas* 2013; 42: 483-487. DOI: 10.1097/MPA.0b013e318267c879.

35. Singh RK, Poddar B, Baronia AK, et al. Audit of patients with severe acute pancreatitis admitted to an intensive care unit. *Indian journal of gastroenterology : official journal of the Indian Society of Gastroenterology* 2012; 31: 243-252. DOI: 10.1007/s12664-012-0205-1.

36. Tran DD, Oe PL, De Fijter CWH, et al. Acute renal failure in patients with acute pancreatitis: prevalence, risk factors, and outcome. *Nephrology, dialysis, transplantation : official publication of the European Dialysis and Transplant Association - European Renal Association* 1993; 8: 1079-1084. DOI: 10.1093/ndt/8.10.1079.

37. Guo Q, Li A, Xia Q, et al. The role of organ failure and infection in necrotizing pancreatitis: a prospective study. *Annals of surgery* 2014; 259: 1201-1207. DOI: 10.1097/sla.0000000000000264.

38. Abraham P, Rodriques J, Moulick N, et al. Efficacy and safety of intravenous ulinastatin versus placebo along with standard supportive care in subjects with mild or severe acute pancreatitis. *Journal of the Association of Physicians of India* 2013; 61: 535-538.

39. Muller CA, Belyaev O, Vogeser M, et al. Corticosteroid-binding globulin: a possible early predictor of infection in acute necrotizing pancreatitis. *Scand J Gastroenterol* 2007; 42: 1354-1361. DOI: 10.1080/00365520701416691.

40. Park HS, In SG, Yoon HJ, et al. Predictive values of neutrophil-lymphocyte ratio as an early indicator for severe acute pancreatitis in the emergency department patients. *J Lab Physicians* 2019; 11: 259-264. DOI: 10.4103/jlp.Jlp_82_19.

41. Podda M, Pacella D, Pellino G, et al. coMpliAnce with evideNce-based cliniCal guidelines in the managemenT of acute biliaRy pancreAtitis): The MANCTRA-1 international audit. *Pancreatology* 2022; 22: 902-916. 20220716. DOI: 10.1016/j.pan.2022.07.007.
